# A Multi-Platform Metabolomics Approach Identifies Urinary Metabolite Signatures That Differentiate Ketotic From Healthy Dairy Cows

**DOI:** 10.3389/fvets.2021.595983

**Published:** 2021-01-26

**Authors:** Guanshi Zhang, Rupasri Mandal, David S. Wishart, Burim N. Ametaj

**Affiliations:** ^1^Department of Agricultural, Food and Nutritional Science, University of Alberta, Edmonton, AB, Canada; ^2^Departments of Biological Sciences and Computing Science, University of Alberta, Edmonton, AB, Canada

**Keywords:** ketosis, dairy cows, metabolomics, biomarkers, urine

## Abstract

Ketosis and subclinical ketosis are widespread among dairy cows especially after calving. Etiopathology of ketosis has been related to negative energy balance. The objective of this study was to investigate metabolite fingerprints in the urine of pre-ketotic, ketotic, and post-ketotic cows to identify potential metabolite alterations that can be used in the future to identify susceptible cows for ketosis and metabolic pathways involved in the development of disease. In this study, NMR, DI/LC-MS/MS, and GC-MS-based metabolomics were used to analyze urine samples from 6 cows diagnosed with ketosis and 20 healthy control (CON) cows at −8 and −4 weeks prepartum, the week (+1 to +3) of ketosis diagnosis, and at +4 and +8 weeks after parturition. Univariate and multivariate analyses were used to screen metabolite panels that can identify cows at their pre-ketotic stage. A total of 54, 42, 48, 16, and 31 differential metabolites between the ketotic and CON cows were identified at −8 and −4 weeks prepartum, ketosis week, and at +4, and +8 weeks postpartum, respectively. Variable importance in projection (VIP) plots ranked the most significant differential metabolites, which differentiated ketotic cows from the CON ones. Additionally, several metabolic pathways that are related to ketosis were identified. Moreover, two promising metabolite panels were identified which clearly separated ketotic from CON cows with excellent level of sensitivity and specificity. Overall, multiple urinary metabolite alterations were identified in pre-ketotic, ketotic, and post-ketotic cows. The metabolite panels identified need to be validated in the future in a larger cohort of animals.

## Introduction

Ketosis is a prevalent metabolic disorder of dairy cattle characterized by increased levels of ketone bodies such as β-hydroxybutyric acid (BHBA), acetoacetate (AcAc), and acetone (Ac) in the blood, milk, and urine during the early lactation (1). Several recent metabolomics articles related to ketosis in dairy cows have been reporting multiple alterations in the blood and milk of the affected cows (2–5). The studies are important contributions to the better understanding of the pathobiology of the disease and its diagnosis. However, it would be of interest to develop new screening or monitoring urinary biomarker panels of ketosis, at the earliest stages, before the clinical disease appears. Also, using urine as an analytical biofluid would be more advantageous because it is non-invasively obtained and easily accessible in large volumes. Additionally, urine has a low content of proteins and lipids, comparable to plasma or serum, that interfere in the analytical process. Metabolomics analysis of urine samples has been used for identifying biomarkers in several human studies (6, 7). To the best of our knowledge, no comprehensive urinary metabolomics and lipidomics profiling for the identification of predictive biomarkers of ketosis has been previously reported in dairy cows.

The current urine biomarker, AcAc, for the diagnosis of ketosis has been widely used by the dairy industry in the form of a semi-quantitative dipstick; however, this is a qualitative test limited by its low sensitivity, and it is used for diagnostic purposes only (8). Unfortunately, diagnosis of ketosis is a late event when the disease process is already advanced and the affected cows need to be immediately medicated with solutions rich in glucose or its precursors. On the other hand, it might be of interest for the dairy industry to use urinary predicative biomarkers in the early ketosis stage so that the disease susceptibility or risk can be predicted and preventing measures can be applied.

Several previous metabolomics studies have shown that cows affected by ketosis, besides the elevated blood and milk ketone bodies, have many other altered metabolites in the same biofluids (2, 3, 5, 9). Similar fluctuations of metabolites/lipids might be present in the urine of affected cows and can be used to identify cows susceptible to ketosis during the earliest stages of disease and even predict the risk of occurrence much earlier than with the current diagnostic tools.

We hypothesized that pre-ketotic, ketotic, and post-ketotic cows might have presence of multiple metabolite/lipid alterations in the urine which can be detected by utilization of a combination of three integrated metabolomics and lipidomics analytical platforms including high resolution nuclear magnetic resonance (NMR) spectroscopy, direct injection/liquid—chromatography tandem mass spectrometry (DI/LC-MS/MS), and gas chromatography-mass spectrometry (GC-MS). Moreover, alterations in the urine metabolites/lipids might appear in the urine starting at −8/−4 weeks prepartum, during diagnosis of disease (+1 to +3 weeks postpartum), and after the disease event at +4/+8 weeks postpartum. Therefore, the objectives of this investigation were to identify presence of alterations of urinary metabolites/lipids in pre-ketotic cows and panels of very highly specific metabolites/lipids that can be used to distinguish pre-ketotic cows from healthy controls (CON) during the dry off period as well as to identify a novel panel of metabolites/lipids that can be used to diagnose ketosis with greater sensitivity and specificity, and monitor progression of disease after treatment.

## Materials and Methods

This study was part of a prospective project designed to identify screening panels of metabolite biomarkers of six periparturient diseases in dairy cows. All experimental procedures were approved by the University of Alberta Animal Policy and Welfare Committee for Livestock, and animals were cared for in accordance with the guidelines of the Canadian Council on Animal Care (10). The metabolomics analyses were performed at the Metabolomics Innovation Center, University of Alberta, Edmonton, AB, Canada.

### Animals and Diets

One hundred pregnant Holstein dairy cows at the Dairy Research and Technology Center, University of Alberta, were used in this study. We excluded all cases with other periparturient diseases during the experimental period. Six multiparous (parity: 3.0 ± 0.6, mean ± SEM) Holstein dairy cows were diagnosed postpartum with ketosis by urinary Ketostix strips (score > “moderate” (3,920 μmol of AcAc/L), Bayer Corporation, Elkhart, IN) and confirmed diagnosis by a colorimetric method [i.e., serum β-hydroxybutyric acid (BHBA) ≥1,400 μmol/L; kit provided by Sigma, St. Louis, MO, USA] in the lab. More precisely, concentrations of BHBA in the serum of CON and ketotic cows were 690 ± 80.17 and 1,756 ± 178 μmol/L (11). Twenty healthy control (CON) cows that were similar in parity (3.1 ± 0.4) and body condition score (BCS) (BCS = 3 after calving), with the other six cows diagnosed with ketosis were selected for this nested case-control study. Detailed information about clinical assessment of animals were previously published (11).

The total experimental period for each cow was 16 weeks, starting from −8 weeks before the expected day of parturition until +8 weeks postpartum. The close-up diet prepartum and the fresh lactation diet for the cows have been previously reported (11). All total mixed ration (TMR) were formulated to meet or exceed the nutrient requirements of a 680 kg lactating cows as per National Research Council (NRC) guidelines (12). All disease information and veterinary treatments were recorded for each cow throughout the experimental period.

### Urine Sample Collection

Urine samples were obtained from 100 transition Holstein dairy cows once per week at 0700 before feeding from −8 weeks prepartum to +8 weeks postpartum. A midstream sample of naturally voided urine sample was collected by gently massaging the perineal area in a 20 mL sterile tube. Fecal material or other debris from exterior of the vulva were removed, prior to sample collection, by washing the area with warm water and soap and then disinfected it with alcohol.

Twenty healthy controls and six cows that developed ketosis were selected for further metabolomics analyses. Urine metabolomics analyses were conducted on samples from five time points at −8 weeks (53–59 days) and −4 weeks (25–31 days) prior to the parturition, the disease (5–21 days, mean: 13 days) week, and at +4 weeks (25–31 days) and +8 weeks (53–59 days) after calving from each cow. Urine samples were stored at −80°C until lab analyses to avoid loss of bioactivity and contamination. All samples were thawed on ice for approximately 2 h before use.

### DI/LC-MS/MS Compound Identification and Quantification

To quantify concentrations of amino acids (AAs), acylcarnitines (ACs), biogenic amines, glycerophospholipids, sphingolipids, and hexose in urine samples, a targeted approach was applied using a commercial kit (Absolute*IDQ* 180, BIOCRATES Life Science AG, Innsbruck, Austria) that combined direct injection and tandem mass spectrometry (DI-MS/MS) with a reverse-phase liquid chromatography and tandem mass spectrometry (LC-MS/MS)-based metabolomics. An ABI 4000 Q-Trap mass spectrometer (Applied Biosystems/MDS Sciex, Foster City, CA) was used for this kit-based targeted analyses. Detailed procedures for derivatization and extraction of analytes, kit preparation, and MS running have been described previously (13).

### NMR Compound Identification and Quantification

All proton NMR (^1^H-NMR) spectra were obtained on a 500 MHz Inova spectrometer (Varian Inc., Palo Alto, CA) equipped with a 5 mm hydrogen, carbon, and nitrogen (HCN) Z-gradient pulsed-field gradient (PFG) Varian cold-probe (14). Chenomx NMR Suite Professional software package (version 7.6, Chenomx Inc., Edmonton, AB, Canada) was applied to profile raw ^1^H-NMR spectra as previously described (15). Details on the NMR urine sample preparation, NMR spectra acquisition, and raw NMR data profiling have been previously reported (16).

### GC-MS Compound Identification and Quantification

The extraction and derivatization protocol for urinary organic acids was optimized based on a previously reported method (17). Derivatized extracts (e.g., organic acids) were injected by an Agilent 7683 Series autosampler (Agilent Technologies, Palo Alto, CA, USA) followed by the analysis employing Agilent 6890N GC system coupled with electron impact (EI) ionization mode 5973N mass selective detector (Agilent Technologies, Palo Alto, CA, USA). Raw GC-MS data (“.D” file format) were first transformed into CDF format by the ChemStation Data Analysis software (Agilent Technologies, Palo Alto, CA, USA) prior to data pretreatment. Identification and quantification of metabolites was firstly processed and analyzed automatically by a web-based software called GC-AutoFit (http://gcms.wishartlab.com/) and results were further confirmed manually following the method as previously described (18). Further details on the organic acid extraction, derivatization, separation, and GC-MS data processing of urine samples were elaborated previously (19).

### Statistical Analysis

All absolute metabolite/lipid concentrations quantified from different analytical approaches were normalized to each urine sample's corresponding creatinine level (assuming a constant rate creatinine excretion for each urine sample) to compensate for variations in the urine volume. The concentration of each metabolite is expressed as μM/mM creatinine. All concentration data from different metabolomics platforms (i.e., DI/LC-MS/MS, NMR, and GC-MS) were pooled together for the multivariate analysis, metabolic pathway analysis, and biomarker analysis.

Considering complicated longitudinal metabolomics datasets and the very large number of variables, the mixed model ANOVA was not used for statistical processing of data. Instead, univariate analysis [i.e., *t*-test or Wilcoxon-Mann-Whitney (rank sum) test] of data was performed to compare concentrations of urinary metabolites at five timepoints, respectively, using R (Version 3.0.3, R Development Core Team, 2008, https://www.r-project.org). Statistical significance was declared at *P* < 0.05. All metabolomics data were processed and analyzed using the MetaboAnalyst software (20). Recommended statistical procedures for metabolomics analyses were followed according to previously published protocols (20). Metabolites that were frequently (>20%) below the limit of detection or with more than 20% missing values were excluded from datasets. Otherwise, missing values were replaced by a value of one-half of the minimum positive value in the original dataset. Data normalization of metabolite concentration was done prior to the statistical analysis, metabolic pathway analysis, and biomarker analysis to create a Gaussian distribution (20). In the current study, we used log-transformed and auto scaling metabolite values.

To perform a standard cross-sectional two-group study, we compared CON cows and cows with ketosis at each time point (−8, −4, disease diagnosis, +4, and +8 weeks around calving), respectively. Principal component analysis (PCA), partial least squares—discriminant analysis (PLS-DA), quantitative enrichment analysis, and metabolic pathway analysis were performed via MetaboAnalyst. In the PLS-DA model, a variable importance in the projection (VIP) plot was used to rank the metabolites based on their importance in discriminating ketotic group from the group of CON cows. Metabolites with the highest VIP values are the most powerful group discriminators. Typically, VIP values >1 are significant and VIP values >2 are highly significant. A 2,000 permutation test was implemented to validate the reliability of the model because it used random resampling of ketotic and CON cows to determine the probability that metabolites distinguishing ketotic and CON groups is not a result of chance.

Urinary biomarkers and the quality of biomarker panels were determined using receiver-operator characteristic (ROC) curves as calculated by MetaboAnalyst 3.0 (21). ROC curves are often summarized into a single metric known as the area under the ROC curve (AUC), which indicates the accuracy of a test for correctly distinguishing one group such as ketotic cows from CON ones. A permutation testing (with 1,000 resampling) was conducted for each ROC curve at different time points.

## Results

The DI/LC-MS/MS targeted metabolomics/lipids analyses quantified 140 metabolites [40 ACs, 9 lysophosphatidylcholine (lysoPC), 67 phosphatidylcholine (PC), 9 hydroxysphingomyelin [SM (OH)] or sphingomyelin (SM), hexose, 11 AAs, and 3 biogenic amines] in the urine of both ketotic and CON groups of cows (Table 1 and Supplementary Tables 1, 2). Eighty-eight metabolites (26 organic acids, 36 AAs and derivatives, 6 saccharides, 3 ketones, 3 alcohols, and 14 misc) were identified and quantified in each urine sample by ^1^H-NMR analysis (Table 1 and Supplementary Tables 3, 4). A total of 54 metabolites (i.e., organic acids) were measured by GC-MS, however, only 13 organic acids were consistently identified and quantified in most urine samples (Table 1 and Supplementary Tables 5, 6). The complete list of metabolite/lipid concentrations [means ± standard deviations (SD)], *P*-value, fold change, and direction of change (up or down) in pre-ketotic cows, cows diagnosed with ketosis, and post-ketotic cows relative to CON cows are shown in Table 1 and Supplementary Tables 1–6. Metabolites detected with more than 20% missing values are not reported in this paper.

**Table 1:** Concentrations of significant urine metabolites [mean (SD)] in healthy control (CON) and ketotic cows at 3 time points (−8, −4 weeks, and the week of diagnosis of disease) as determined by DI/LC-MS/MS, NMR, and GC-MS.

**Metabolite, μM/mM creatinine^b, c^**	**8 weeks before parturition**					**4 weeks before parturition**					**Ketosis diagnosis week^a^**				
	**Ketosis**	**CON**	***P*-value^d^**	**Fold change**	**Ketosis/CON**	**Ketosis**	**CON**	***P*-value**	**Fold change**	**Ketosis/CON**	**Ketosis**	**CON**	***P*-value**	**Fold change**	**Ketosis/CON**
**Number of cases**	**6**	**20**	**-**	**-**	**-**	**6**	**20**	**-**	**-**	**-**	**6**	**20**	**-**	**-**	
**Acylcarnitines^e^**															
C10:1	0.055 (0.037)	0.027 (0.009)	0.0029 (W)	2.05	Up	0.077 (0.075)	0.031 (0.012)	0.0029 (W)	2.48	Up	0.038 (0.013)	0.025 (0.012)	0.0668 (W)	1.52	Up
C10:2	0.019 (0.008)	0.010 (0.004)	0.0043 (W)	1.82	Up	0.022 (0.016)	0.010 (0.007)	0.0107 (W)	2.2	Up	0.010 (0.003)	0.012 (0.007)	0.9764 (W)	−1.13	Down
C12	0.138 (0.222)	0.028 (0.017)	0.0949 (W)	4.95	Up	0.146 (0.286)	0.024 (0.020)	0.2185 (W)	6.05	Up	0.158 (0.115)	0.053 (0.036)	0.0011 (W)	2.99	Up
C12-DC	0.027 (0.010)	0.018 (0.006)	0.0093 (W)	1.52	Up	0.032 (0.026)	0.017 (0.004)	0.2464 (W)	1.88	Up	0.026 (0.014)	0.016 (0.005)	0.0765 (W)	1.63	Up
C14:1	0.004 (0.003)	0.003 (0.001)	0.0391 (W)	1.75	Up	0.011 (0.013)	0.003 (0.002)	0.0008 (W)	4.09	Up	0.005 (0.002)	0.004 (0.002)	0.0720 (W)	1.3	Up
C14:1-OH	0.007 (0.002)	0.003 (0.001)	0.0002 (W)	2.27	Up	0.007 (0.009)	0.003 (0.002)	0.3875 (W)	2.11	Up	0.007 (0.003)	0.003 (0.002)	0.0034 (W)	2.14	Up
C14:2-OH	0.005 (0.001)	0.003 (0.001)	0.0077	1.56	Up	0.025 (0.043)	0.004 (0.005)	0.0331 (W)	5.64	Up	0.010 (0.008)	0.006 (0.006)	0.3244 (W)	1.56	Up
C16	0.013 (0.007)	0.004 (0.002)	0.0001 (W)	3.35	Up	0.012 (0.008)	0.004 (0.002)	0.0055 (W)	3.31	Up	0.008 (0.004)	0.004 (0.002)	0.0069 (W)	2.2	Up
C16-OH	0.006 (0.004)	0.002 (0.001)	0.0004 (W)	2.62	Up	0.006 (0.006)	0.003 (0.002)	0.1963 (W)	2.25	Up	0.005 (0.001)	0.003 (0.002)	0.0062	1.73	Up
C16:1	0.013 (0.004)	0.007 (0.003)	0.0006 (W)	2.06	Up	0.023 (0.030)	0.007 (0.005)	0.0279 (W)	3.28	Up	0.011 (0.007)	0.008 (0.004)	0.6999 (W)	1.27	Up
C16:2	0.005 (0.002)	0.002 (0.001)	0.018	2.63	Up	0.006 (0.006)	0.002 (0.001)	0.0055 (W)	2.94	Up	0.004 (0.002)	0.002 (0.001)	0.0043 (W)	1.94	Up
C18	0.006 (0.005)	0.003 (0.001)	0.0417 (W)	2.4	Up	0.006 (0.004)	0.003 (0.001)	0.0133 (W)	2.48	Up	0.004 (0.002)	0.002 (0.001)	0.0580 (W)	1.81	Up
C18:1	0.008 (0.006)	0.004 (0.002)	0.0261 (W)	1.76	Up	0.006 (0.004)	0.004 (0.001)	0.7304 (W)	1.66	Up	0.005 (0.002)	0.004 (0.002)	0.0996 (W)	1.28	Up
C18:1-OH	0.010 (0.011)	0.005 (0.002)	0.1101 (W)	2.12	Up	0.008 (0.006)	0.004 (0.001)	0.0358 (W)	2.03	Up	0.008 (0.006)	0.005 (0.003)	0.4373 (W)	1.65	Up
C18:2	0.011 (0.009)	0.002 (0.001)	0.0619	5.84	Up	0.008 (0.008)	0.002 (0.001)	0.0020 (W)	4.51	Up	0.007 (0.003)	0.002 (0.002)	0.0279 (W)	3.2	Up
C3-OH	0.048 (0.026)	0.010 (0.006)	0.0152	4.59	Up	0.049 (0.031)	0.010 (0.005)	0.0257	4.92	Up	0.025 (0.021)	0.013 (0.009)	0.0279 (W)	1.89	Up
C3:1	0.010 (0.005)	0.021 (0.009)	0.0069 (W)	−1.99	Down	0.021 (0.026)	0.022 (0.012)	0.0622 (W)	−1.02	Down	0.011 (0.005)	0.027 (0.018)	0.0015 (W)	−2.51	Down
C5-M-DC	0.054 (0.033)	0.012 (0.005)	<0.0001 (W)	4.38	Up	0.054 (0.053)	0.014 (0.009)	0.0008 (W)	3.95	Up	0.053 (0.018)	0.017 (0.011)	<0.0001 (W)	3.15	Up
C5-OH (C3-DC-M)	0.056 (0.026)	0.024 (0.007)	0.0046 (W)	2.33	Up	0.069 (0.065)	0.027 (0.015)	0.0160 (W)	2.52	Up	0.058 (0.011)	0.036 (0.025)	0.0017 (W)	1.63	Up
C5-DC (C6-OH)	0.016 (0.006)	0.010 (0.004)	0.0033	1.61	Up	0.028 (0.037)	0.011 (0.006)	0.0949 (W)	2.56	Up	0.021 (0.005)	0.015 (0.011)	0.0069 (W)	1.47	Up
C6:1	0.020 (0.010)	0.012 (0.005)	0.0026 (W)	1.75	Up	0.027 (0.031)	0.012 (0.006)	0.1082 (W)	2.2	Up	0.017 (0.007)	0.012 (0.007)	0.1082 (W)	1.37	Up
C7-DC	0.015 (0.005)	0.007 (0.003)	<0.0001	2.07	Up	0.017 (0.017)	0.007 (0.004)	0.0131 (W)	2.42	Up	0.014 (0.003)	0.007 (0.005)	0.0008 (W)	1.99	Up
C8	0.036 (0.022)	0.020 (0.006)	0.0307 (W)	1.74	Up	0.035 (0.028)	0.019 (0.005)	0.2729 (W)	1.84	Up	0.020 (0.007)	0.017 (0.005)	0.2256 (W)	1.17	Up
**Lysophosphatidylcholines^e^**															
lysoPC a C18:2	0.007 (0.011)	0.009 (0.005)	0.2215 (W)	−1.27	Down	0.007 (0.007)	0.023 (0.041)	0.0261 (W)	−3.12	Down	0.008 (0.005)	0.013 (0.015)	0.5549 (W)	−1.63	Down
lysoPC a C28:1	0.004 (0.009)	0.003 (0.002)	0.0470 (W)	1.26	Up	0.002 (0.004)	0.003 (0.006)	0.2822 (W)	−1.51	Down	0.003 (0.002)	0.003 (0.002)	0.4748 (W)	−1.28	Down
**Phosphatidylcholines^e^**															
PC aa C28:1	0.011 (0.006)	0.009 (0.003)	0.0358 (W)	1.32	Up	0.019 (0.019)	0.013 (0.010)	0.8755 (W)	1.49	Up	0.011 (0.003)	0.008 (0.002)	0.1442 (W)	1.28	Up
PC aa C32:0	0.004 (0.004)	0.004 (0.003)	0.6384 (W)	1.09	Up	0.002 (0.002)	0.024 (0.055)	0.0188 (W)	−10.07	Down	0.002 (0.005)	0.003 (0.005)	0.0399 (W)	−1.39	Down
PC aa C32:2	0.003 (0.003)	0.007 (0.006)	0.0261 (W)	−2.33	Down	0.002 (0.002)	0.010 (0.012)	0.0013 (W)	−4.88	Down	0.002 (0.002)	0.006 (0.003)	0.0047 (W)	−2.39	Down
PC aa C34:1	0.009 (0.008)	0.036 (0.071)	0.2185 (W)	−4.17	Down	0.005 (0.009)	0.194 (0.762)	0.0279 (W)	−36.47	Down	0.014 (0.019)	0.042 (0.085)	0.6565 (W)	−3.04	Down
PC aa C34:2	0.013 (0.025)	0.090 (0.203)	0.0131 (W)	−7.1	Down	0.012 (0.011)	0.432 (1.700)	0.0622 (W)	−37.38	Down	0.012 (0.014)	0.094 (0.188)	0.1565 (W)	−7.87	Down
PC aa C34:3	0.001 (0.001)	0.006 (0.015)	0.3767 (W)	−5.18	Down	0.001 (0.002)	0.016 (0.060)	0.4424 (W)	−13.41	Down	0.001 (0.001)	0.006 (0.013)	0.0297 (W)	−9.73	Down
PC aa C34:4	0.001 (0.002)	0.003 (0.002)	0.0413 (W)	−2.73	Down	0.002 (0.002)	0.004 (0.007)	0.8312 (W)	−1.78	Down	0.001 (0.001)	0.004 (0.004)	0.0720 (W)	−3.07	Down
PC aa C36:0	0.052 (0.030)	0.030 (0.012)	0.0417 (W)	1.73	Up	0.049 (0.028)	0.032 (0.019)	0.0742 (W)	1.52	Up	0.023 (0.011)	0.021 (0.005)	0.7325 (W)	1.1	Up
PC aa C36:3	0.004 (0.008)	0.022 (0.048)	0.0161 (W)	−5.88	Down	0.004 (0.004)	0.103 (0.395)	0.1756 (W)	−24.22	Down	0.003 (0.004)	0.028 (0.055)	0.0622 (W)	−8.56	Down
PC aa C36:4	0.005 (0.004)	0.031 (0.064)	0.1229 (W)	−6.37	Down	0.005 (0.007)	0.293 (1.238)	0.0459 (W)	−56.84	Down	0.004 (0.005)	0.031 (0.057)	0.0551 (W)	−8.6	Down
PC aa C36:5	0.001 (0.002)	0.003 (0.007)	0.0159 (W)	−5.01	Down	0.001 (0.001)	0.025 (0.102)	0.1185 (W)	−28.39	Down	0.002 (0.003)	0.003 (0.006)	0.3294 (W)	−2	Down
PC aa C38:0	0.005 (0.002)	0.003 (0.001)	0.0484 (W)	1.42	Up	0.005 (0.006)	0.009 (0.016)	0.1586 (W)	−1.8	Down	0.003 (0.002)	0.003 (0.002)	0.4015 (W)	−1	Down
PC aa C38:3	0.005 (0.005)	0.005 (0.004)	0.3977 (W)	−1.03	Down	0.004 (0.006)	0.050 (0.125)	0.0188 (W)	−14.16	Down	0.001 (0.001)	0.008 (0.015)	0.0069 (W)	−7.61	Down
PC aa C38:5	0.001 (0.001)	0.010 (0.020)	0.0026 (W)	−17.47	Down	0.001 (0.002)	0.041 (0.164)	0.0931 (W)	−40.06	Down	0.002 (0.003)	0.010 (0.020)	0.1130 (W)	−4.92	Down
PC aa C40:1	0.035 (0.013)	0.033 (0.011)	0.6838 (W)	1.07	Up	0.050 (0.041)	0.034 (0.010)	0.8755 (W)	1.48	Up	0.019 (0.009)	0.031 (0.012)	0.0274 (W)	−1.63	Down
PC aa C42:0	0.006 (0.011)	0.005 (0.002)	0.0417 (W)	1.21	Up	0.005 (0.002)	0.004 (0.003)	0.4712 (W)	1.2	Up	0.002 (0.001)	0.003 (0.002)	0.1442 (W)	−1.34	Down
PC aa C42:4	0.002 (0.003)	0.001 (0.000)	0.5099 (W)	2.52	Up	0.001 (0.001)	0.001 (0.002)	0.0357 (W)	−2.42	Down	0.001 (0.001)	0.001 (0.001)	0.9752 (W)	1.19	Up
PC aa C42:5	0.001 (0.001)	0.001 (0.001)	0.5722 (W)	−1.4	Down	0.009 (0.006)	0.004 (0.003)	0.0283 (W)	2.06	Up	0.008 (0.005)	0.003 (0.002)	0.0433 (W)	2.58	Up
PC aa C42:6	0.057 (0.035)	0.027 (0.010)	0.0307 (W)	2.16	Up	0.058 (0.056)	0.024 (0.009)	0.1409 (W)	2.39	Up	0.026 (0.014)	0.019 (0.005)	0.4373 (W)	1.4	Up
PC ae C30:2	0.004 (0.003)	0.011 (0.012)	0.0560 (W)	−3.02	Down	0.002 (0.002)	0.012 (0.011)	0.0036 (W)	−7.71	Down	0.000 (0.001)	0.016 (0.016)	0.0017 (W)	−35.06	Down
PC ae C36:0	0.011 (0.010)	0.009 (0.004)	0.9251 (W)	1.2	Up	0.011 (0.011)	0.010 (0.005)	0.4712 (W)	1.11	Up	0.004 (0.002)	0.009 (0.004)	0.0057 (W)	−2.04	Down
PC ae C36:1	0.009 (0.007)	0.004 (0.004)	0.0391 (W)	2.2	Up	0.005 (0.003)	0.012 (0.041)	0.2681 (W)	−2.75	Down	0.003 (0.002)	0.006 (0.006)	0.6999 (W)	−1.66	Down
PC ae C36:2	0.001 (0.001)	0.006 (0.008)	0.0056 (W)	−4.39	Down	0.004 (0.006)	0.017 (0.051)	0.4839 (W)	−3.93	Down	0.002 (0.002)	0.007 (0.010)	0.1756 (W)	−3.29	Down
PC ae C36:3	0.004 (0.003)	0.002 (0.004)	0.0282 (W)	2.25	Up	0.001 (0.003)	0.006 (0.022)	0.2005 (W)	−4.64	Down	0.001 (0.001)	0.003 (0.005)	0.7377 (W)	−2.8	Down
PC ae C36:4	0.008 (0.002)	0.004 (0.004)	0.0020 (W)	2.01	Up	0.015 (0.013)	0.020 (0.073)	0.0087 (W)	−1.33	Down	0.009 (0.004)	0.004 (0.005)	0.0194 (W)	2.03	Up
PC ae C38:0	0.048 (0.035)	0.023 (0.008)	0.0484 (W)	2.12	Up	0.037 (0.030)	0.021 (0.012)	0.5944 (W)	1.73	Up	0.022 (0.011)	0.017 (0.008)	0.1130 (W)	1.3	Up
PC ae C38:6	0.008 (0.005)	0.005 (0.003)	0.1229 (W)	1.56	Up	0.014 (0.010)	0.013 (0.035)	0.0160 (W)	1.1	Up	0.010 (0.012)	0.005 (0.005)	0.4942 (W)	2.05	Up
PC ae C42:0	0.054 (0.043)	0.090 (0.043)	0.0858	−1.66	Down	0.074 (0.046)	0.097 (0.097)	0.5727 (W)	−1.32	Down	0.031 (0.015)	0.105 (0.088)	0.0043 (W)	−3.37	Down
PC ae C42:1	0.003 (0.003)	0.008 (0.004)	0.0053	−2.89	Down	0.004 (0.006)	0.008 (0.007)	0.1906 (W)	−1.96	Down	0.002 (0.002)	0.008 (0.008)	0.0055 (W)	−4.06	Down
PC ae C42:5	0.080 (0.040)	0.077 (0.033)	0.8755 (W)	1.04	Up	0.067 (0.059)	0.070 (0.022)	0.2218 (W)	−1.04	Down	0.038 (0.015)	0.072 (0.037)	0.0069 (W)	−1.87	Down
PC ae C44:3	0.005 (0.003)	0.002 (0.002)	0.0107 (W)	2.16	Up	0.010 (0.009)	0.003 (0.003)	0.0026 (W)	4.01	Up	0.007 (0.005)	0.002 (0.002)	0.0279 (W)	3.16	Up
PC ae C44:6	0.002 (0.001)	0.003 (0.002)	0.0969 (W)	−1.53	Down	0.001 (0.002)	0.003 (0.004)	0.1513 (W)	−2.42	Down	0.000 (0.001)	0.002 (0.002)	0.0023 (W)	−6.81	Down
**Sphingomyelins^e^**															
SM (OH) C22:1	0.001 (0.001)	0.001 (0.002)	0.0470 (W)	−2.68	Down						0.003 (0.006)	0.003 (0.004)	0.5140 (W)	1.2	Up
SM C16:0	0.005 (0.008)	0.020 (0.037)	0.0081 (W)	−4.1	Down	0.004 (0.007)	0.134 (0.544)	0.0552 (W)	−31.09	Down	0.004 (0.005)	0.021 (0.042)	0.3152 (W)	−4.77	Down
Hexoses	586.956 (892.842)	167.809 (136.369)	0.0536 (W)	3.5	Up	595.632 (1122.887)	127.923 (107.469)	0.1963 (W)	4.66	Up	676.828 (440.753)	317.042 (251.094)	0.0107 (W)	2.13	Up
**Amino acids^e^**															
L-Arginine	4.236 (2.082)	1.660 (0.929)	0.0004 (W)	2.55	Up	3.661 (1.977)	1.625 (1.033)	0.0526	2.25	Up	2.830 (0.548)	2.232 (1.677)	0.1082 (W)	1.27	Up
L-Aspartic acid	12.116 (1.923)	5.403 (3.316)	<0.0001	2.24	Up	13.100 (8.473)	5.547 (4.454)	0.0043 (W)	2.36	Up	10.777 (7.565)	9.257 (6.956)	0.5727 (W)	1.16	Up
L-Glutamine	72.655 (53.188)	34.293 (18.939)	0.0194 (W)	2.12	Up	68.891 (40.520)	40.401 (24.753)	0.1229 (W)	1.71	Up	49.503 (14.022)	42.708 (33.225)	0.2425 (W)	1.16	Up
Glycine	77.556 (86.769)	20.064 (11.634)	0.0087 (W)	3.87	Up	44.987 (31.625)	32.120 (40.947)	0.1229 (W)	1.4	Up	142.226 (151.547)	109.707 (137.548)	0.4215 (W)	1.3	Up
L-Threonine	19.195 (5.669)	10.845 (4.950)	0.0018	1.77	Up	22.528 (18.132)	11.791 (7.553)	0.0949 (W)	1.91	Up	10.600 (4.422)	11.141 (7.550)	0.7897 (W)	−1.05	Down
L-Tryptophan	7.153 (2.704)	2.864 (1.516)	0.0002 (W)	2.5	Up	8.536 (6.344)	3.023 (2.310)	0.0055 (W)	2.82	Up	4.205 (2.730)	2.948 (2.211)	0.1963 (W)	1.43	Up
**Biogenic amines^e^**															
ADMA	6.074 (1.405)	3.576 (1.985)	0.0087	1.7	Up	5.519 (4.123)	3.139 (2.356)	0.0459 (W)	1.76	Up	1.458 (0.779)	1.528 (1.488)	0.4942 (W)	−1.05	Down
SDMA	13.196 (10.632)	4.535 (1.556)	<0.0001 (W)	2.91	Up	25.891 (35.428)	5.072 (2.504)	0.0536 (W)	5.1	Up	19.125 (18.576)	4.899 (3.686)	0.0011 (W)	3.9	Up
Carnosine	2.434 (0.637)	1.226 (0.467)	<0.0001	1.99	Up	2.700 (1.879)	1.431 (0.943)	0.0194 (W)	1.89	Up	1.718 (1.137)	1.424 (1.318)	0.3244 (W)	1.21	Up
**Ketones^f^**															
3-Hydroxybutyric acid	25.246 (11.676)	28.375 (15.580)	0.9764 (W)	−1.12	Down	25.217 (18.953)	19.812 (10.528)	0.6140 (W)	1.27	Up	69.267 (40.517)	38.512 (22.349)	0.0297 (W)	1.8	Up
Acetoacetic acid	10.753 (4.476)	17.634 (11.278)	0.0828 (W)	−1.64	Down	15.925 (12.055)	12.791 (6.194)	0.9764 (W)	1.25	Up	50.235 (27.826)	24.091 (12.914)	0.0067 (W)	2.09	Up
**Saccharides^f^**															
1,3-Dihydroxyacetone (DHA)	9.582 (9.879)	7.568 (8.954)	0.5327 (W)	1.27	Up	3.214 (2.478)	2.728 (3.441)	0.2185 (W)	1.18	Up	23.284 (21.716)	9.103 (9.578)	0.0087 (W)	2.56	Up
Arabinose	48.045 (57.689)	21.320 (13.284)	0.2185 (W)	2.25	Up	46.787 (44.443)	18.660 (15.938)	0.0331 (W)	2.51	Up	26.045 (10.066)	23.691 (17.741)	0.3244 (W)	1.1	Up
D-Galactose	35.230 (16.184)	49.648 (29.686)	0.2681 (W)	−1.41	Down	35.394 (22.728)	42.679 (23.832)	0.2954 (W)	−1.21	Down	16.774 (14.061)	59.357 (105.344)	0.0279 (W)	−3.54	Down
**Amino acids and derivatives^f^**															
3-Aminoisobutyric acid	21.187 (7.816)	16.440 (6.346)	0.1398	1.29	Up	20.879 (7.638)	13.072 (4.522)	0.0043	1.6	Up	20.562 (5.313)	20.129 (11.004)	0.5327 (W)	1.02	Up
beta-Alanine	15.173 (8.445)	7.915 (7.296)	0.0131 (W)	1.92	Up	19.299 (9.514)	7.701 (4.566)	0.0294	2.51	Up	12.743 (10.833)	10.220 (7.205)	0.5327 (W)	1.25	Up
L-Alloisoleucine	14.585 (5.215)	10.751 (8.089)	0.1390 (W)	1.36	Up	18.740 (9.165)	7.821 (5.209)	0.0107 (W)	2.4	Up	16.154 (8.808)	13.769 (11.500)	0.3551 (W)	1.17	Up
L-Cysteine	18.436 (16.806)	9.092 (4.899)	0.1082 (W)	2.03	Up	22.031 (16.731)	10.311 (9.118)	0.0391 (W)	2.14	Up	23.630 (24.107)	11.181 (6.476)	0.1082 (W)	2.11	Up
L-Isoleucine	62.101 (27.536)	39.322 (13.743)	0.1004	1.58	Up	64.746 (19.874)	35.163 (14.131)	0.0011 (W)	1.84	Up	79.777 (77.860)	55.395 (21.891)	0.7897 (W)	1.44	Up
L-Lysine	13.778 (5.475)	5.495 (2.685)	0.0126	2.51	Up	12.861 (5.305)	4.388 (2.224)	0.0103	2.93	Up	14.509 (10.120)	9.930 (11.685)	0.1756 (W)	1.46	Up
L-Phenylalanine	14.885 (8.412)	9.585 (3.470)	0.0720 (W)	1.55	Up	16.583 (8.415)	6.817 (3.047)	<0.0001 (W)	2.43	Up	13.353 (4.837)	12.943 (9.023)	0.4215 (W)	1.03	Up
L-Tyrosine	27.640 (11.445)	15.146 (8.427)	0.0072	1.82	Up	27.903 (8.230)	15.564 (8.026)	0.0031	1.79	Up	22.204 (17.498)	19.355 (14.687)	0.8823 (W)	1.15	Up
L-Valine	1820.749 (670.094)	6181.864 (5171.103)	0.1565 (W)	−3.4	Down	1923.557 (1183.203)	6035.988 (4370.962)	0.0720 (W)	−3.14	Down	1529.750 (696.721)	7893.428 (6576.605)	0.0004	−5.16	Down
N-Acetylaspartic acid	2.984 (0.746)	4.079 (1.924)	0.0498	−1.37	Down	3.165 (3.944)	4.019 (3.176)	0.0720 (W)	−1.27	Down	2.357 (3.343)	4.575 (3.250)	0.0160 (W)	−1.94	Down
N-Acetylglutamic acid	17.363 (4.966)	10.066 (4.351)	0.0019	1.72	Up	22.644 (7.688)	8.507 (4.424)	<0.0001	2.66	Up	10.760 (7.199)	12.654 (7.851)	0.6565 (W)	−1.18	Down
Pantothenic acid	3.415 (2.679)	1.440 (1.410)	0.0391 (W)	2.37	Up	2.900 (1.791)	1.256 (2.306)	0.0055 (W)	2.31	Up	3.036 (1.743)	1.544 (1.993)	0.0279 (W)	1.97	Up
Tiglylglycine	9.940 (5.280)	10.494 (5.263)	0.8358 (W)	−1.06	Down	9.656 (3.332)	6.576 (2.041)	0.0101	1.47	Up	8.721 (4.145)	8.895 (5.407)	0.8823 (W)	−1.02	Down
1-Methylhistidine	9.932 (5.811)	7.158 (5.364)	0.1229 (W)	1.39	Up	9.925 (4.300)	4.813 (2.641)	0.0015 (W)	2.06	Up	9.006 (5.350)	9.724 (6.695)	0.8125	−1.08	Down
3-Methylhistidine	19.731 (8.209)	13.075 (8.092)	0.0536 (W)	1.51	Up	17.892 (3.015)	12.466 (6.255)	0.0194 (W)	1.44	Up	20.058 (17.692)	17.129 (9.769)	0.7897 (W)	1.17	Up
**Organic acids^f^**															
2-Hydroxybutyric acid	34.249 (6.275)	41.201 (15.927)	0.6140 (W)	−1.2	Down	42.609 (15.504)	29.577 (9.454)	0.0177	1.44	Up	48.803 (14.901)	49.176 (28.044)	0.6140 (W)	−1.01	Down
2-Methylglutaric acid	19.479 (11.159)	16.175 (6.067)	0.7445 (W)	1.2	Up	26.165 (13.338)	15.143 (6.664)	0.0391 (W)	1.73	Up	23.969 (13.911)	21.621 (9.483)	0.6373	1.11	Up
Ascorbic acid	25.449 (22.066)	11.883 (6.338)	0.0194 (W)	2.14	Up	16.923 (9.777)	11.613 (6.159)	0.1565 (W)	1.46	Up	20.464 (8.747)	11.719 (5.971)	0.0093	1.75	Up
Gluconic acid	18.197 (20.052)	6.641 (4.856)	0.0331 (W)	2.74	Up	13.263 (10.419)	7.594 (7.516)	0.1082 (W)	1.75	Up	11.107 (5.246)	7.161 (5.194)	0.1565 (W)	1.55	Up
Isocitric acid	37.433 (15.832)	24.020 (9.660)	0.017	1.56	Up	33.832 (9.635)	31.295 (14.421)	0.6914	1.08	Up	21.064 (8.441)	31.523 (14.082)	0.0994	−1.5	Down
L-Lactic acid	10.297 (4.924)	10.025 (3.577)	0.3875 (W)	1.03	Up	11.110 (6.566)	7.514 (3.153)	0.1565 (W)	1.48	Up	11.526 (1.713)	9.184 (2.243)	0.0275	1.26	Up
**Alcohols^f^**															
Methanol	34.202 (23.902)	18.444 (7.778)	0.0459 (W)	1.85	Up	44.579 (21.449)	15.843 (6.796)	<0.0001 (W)	2.81	Up	30.583 (14.662)	24.976 (14.654)	0.3551 (W)	1.22	Up
**Misc^f^**															
3-Indoxyl sulfate	40.896 (17.259)	77.506 (48.890)	0.0331 (W)	−1.9	Down	73.067 (38.848)	51.167 (23.789)	0.1013	1.43	Up	49.885 (28.945)	94.579 (68.913)	0.1963 (W)	−1.9	Down
Creatinine^h^	4039.620 (1860.974)	4723.310 (1621.231)	0.389	−1.17	Down	4060.483 (2160.656)	5024.240 (1519.214)	0.2279	−1.24	Down	7045.717 (3357.020)	4984.760 (2670.551)	0.1963 (W)	1.41	Up
Dimethyl sulfone	103.975 (69.952)	129.998 (60.236)	0.2681 (W)	−1.25	Down	113.746 (83.981)	135.866 (130.289)	0.4215 (W)	−1.19	Down	56.398 (27.406)	150.634 (123.789)	0.0087 (W)	−2.67	Down
Hypoxanthine	33.484 (12.170)	19.818 (9.909)	0.0095	1.69	Up	26.445 (6.114)	15.358 (5.047)	0.0001	1.72	Up	21.763 (7.517)	22.664 (10.565)	0.8482	−1.04	Down
Imidazole	5.496 (3.620)	1.775 (1.127)	0.053	3.1	Up	4.192 (2.976)	1.730 (1.378)	0.0008 (W)	2.42	Up	2.637 (1.633)	1.770 (2.208)	0.1082 (W)	1.49	Up
myo-Inositol	31.890 (23.131)	10.727 (7.825)	0.0020 (W)	2.97	Up	26.347 (10.136)	9.169 (5.178)	0.0001 (W)	2.87	Up	21.854 (9.242)	13.256 (8.300)	0.04	1.65	Up
O-Phosphocholine	103.082 (34.477)	297.378 (583.481)	0.1756 (W)	−2.88	Down	112.245 (40.298)	157.265 (122.158)	0.4942 (W)	−1.4	Down	90.645 (56.855)	330.677 (537.163)	0.0459 (W)	−3.65	Down
Trimethylamine	22.359 (8.931)	22.730 (20.018)	0.3875 (W)	−1.02	Down	22.528 (6.068)	15.265 (7.728)	0.046	1.48	Up	51.183 (28.844)	46.590 (36.616)	0.4570 (W)	1.1	Up
Uracil	15.186 (7.734)	15.405 (5.471)	0.4942 (W)	−1.01	Down	14.526 (14.033)	13.066 (5.051)	0.5727 (W)	1.11	Up	13.780 (5.571)	24.442 (10.664)	0.0284	−1.77	Down
Urea	41.789 (30.263)	146.549 (99.053)	0.0004	−3.51	Down	44.508 (21.820)	144.302 (96.802)	0.0391 (W)	−3.24	Down	43.771 (18.384)	194.566 (153.121)	0.0003	−4.45	Down
**Organic acids^g^**															
Azelaic acid	0.052 (0.056)	0.045 (0.050)	0.6922 (W)	1.16	Up	0.009 (0.014)	0.044 (0.043)	0.0101 (W)	−5.07	Down	0.032 (0.038)	0.038 (0.034)	0.4467 (W)	−1.2	Down

a *Cows were diagnosed with ketosis (n = 6) ranging from week +1 to +3*.

b *C10:1, decenoyl-L-carnitine; C10:2, decadienyl-L-carnitine; C12, dodecanoyl-L-carnitine; C12-DC, dodecanedioyl-L-carnitine; C14:1, tetradecenoyl-L-carnitine; C14:1-OH, hydroxytetradecenoyl-L-carnitine; 14:2-OH, hydroxytetradecadienyl-L-carnitine; C16, hexadecanoyl-L-carnitine; C16-OH, hydroxyhexadecanoyl-L-carnitine; C16:1, hexadecenoyl-L-carnitine; C16:2, hexadecadienyl-L-carnitine; C18, octadecanoyl-L-carnitine; C18:1, octadecenoyl-L-carnitine; C18:1-OH, hydroxyoctadecenoyl-L-carnitine; C18:2, octadecadienyl-L-carnitine; C3-OH, hydroxypropionyl-L-carnitine; C3:1, propenyl-L-carnitine; C5-M-DC, methylglutaryl-L-carnitine; C5-OH (C3-DC-M), methylmalonyl-L-carnitine/Hydroxyvaleryl-L-carnitine; C5-DC (C6-OH), glutaryl-L-carnitine/Hydroxyhexanoyl-L-carnitine; C6:1, hexenoyl-L-carnitine; C7-DC, pimelyl-L-carnitine; C8, octanoyl-L-carnitine; C9, nonayl-L-carnitine; lysoPC a, lysophosphatidylcholine acyl; PC aa, phosphatidylcholine diacyl; PC ae, phosphatidylcholine acyl-alkyl; SM (OH), hydroxysphingomyelin; SM, sphingomyelin; ADMA, asymmetric dimethylarginine; SDMA, symmetric dimethylarginine*.

c *Only significant metabolites (at least at one time point) are listed in the table*.

d *p-value is calculated with t-test as a default, p-value with (W) is calculated by the Wilcoxon Mann Whitney test*.

e *Metabolites measured by DI/LC-MS/MS*.

f *Metabolites uniquely measured by NMR. Metabolites that were also measured by DI/LC-MS/MS and GC-MS are not repeatedly shown (No significant differences among different platforms)*.

g *Metabolites uniquely measured by GC-MS. Metabolites that were also measured by DI/LC-MS/MS and NMR are not repeatedly shown (No significant differences among different platforms)*.

h *Concentration of metabolite (Mean ± SD) is expressed by μM*.

### Urinary Metabolite Fingerprints Preceding Ketosis

Univariate and multivariate analyses were performed to compare urinary metabolite fingerprints of pre-ketotic cows vs. CON cows at two time points during the dry-off period (i.e., −8 and −4 weeks prepartum) to identify predictive biomarkers for ketosis. Univariate analysis (i.e., *t*-test or Wilcoxon Mann Whitney test) indicated 54 and 42 urinary metabolites were altered (*P* < 0.05) in pre-ketosis cows at −8 and −4 weeks, respectively (Table 1). Specifically, the majority of metabolites were elevated in pre-ketotic cows including 20 ACs [e.g., methylglutaryl-L-carnitine (C5-M-DC), octadecadienyl-L-carnitine (C18:2), and hydroxypropionyl-L-carnitine (C3-OH)], 1 lysoPC [i.e., lysophosphatidylcholine acyl C28:1 (lysoPC a C28:1)], 10 PCs [e.g., phosphatidylcholine diacyl C28:1 (PC aa C28:1), PC aa C42:6, and phosphatidylcholine acyl-alkyl C38:0 (PC ae C38:0)], hexose, 12 AAs or derivatives [e.g., L-lysine (L-Lys), L-tryptophan (L-Trp), and pantothenic acid], 3 biogenic amines (BA) [i.e., symmetric dimethylarginine (SDMA), asymmetric dimethylarginine (ADMA), and carnosine], 3 organic acids (i.e., ascorbic acid, gluconic acid, and isocitric acid), 1 alcohol (i.e., methanol), 3 from the misc group (i.e., hypoxanthine, imidazole, and myo-inositol) at −8 weeks, and 13 ACs [e.g., C3-OH, Hexadecanoyl-L-carnitine (C16), and C18:2], 3 PCs (i.e., PC aa C42:5, PC ae C38:6, and PC ae C44:3), 19 AAs or derivatives [e.g., L-phenylalanine (L-Phe), L-Lys, and L-arginine (L-Arg)], 3 BA (i.e., SDMA, ADMA, and carnosine), 1 alcohol (methonal), 3 from the misc group (e.g., myo-inositol, hypoxanthine, and trimethylamine) at −4 weeks (Table 1). Additionally, a few urinary lipids, most of which belong to PCs, were lower in pre-ketotic cows. Examples of this class of lipids include PC aa C32:2 and PC ae C30:2, which were decreased in the urine of pre-ketotic cows compared with CON cows at both −8 and −4 weeks (Table 1). Another two interesting metabolites/lipids were SM C16:0 and urea, both of which decreased in pre-ketotic cows at both time points during the dry off period.

**Figure 1:**
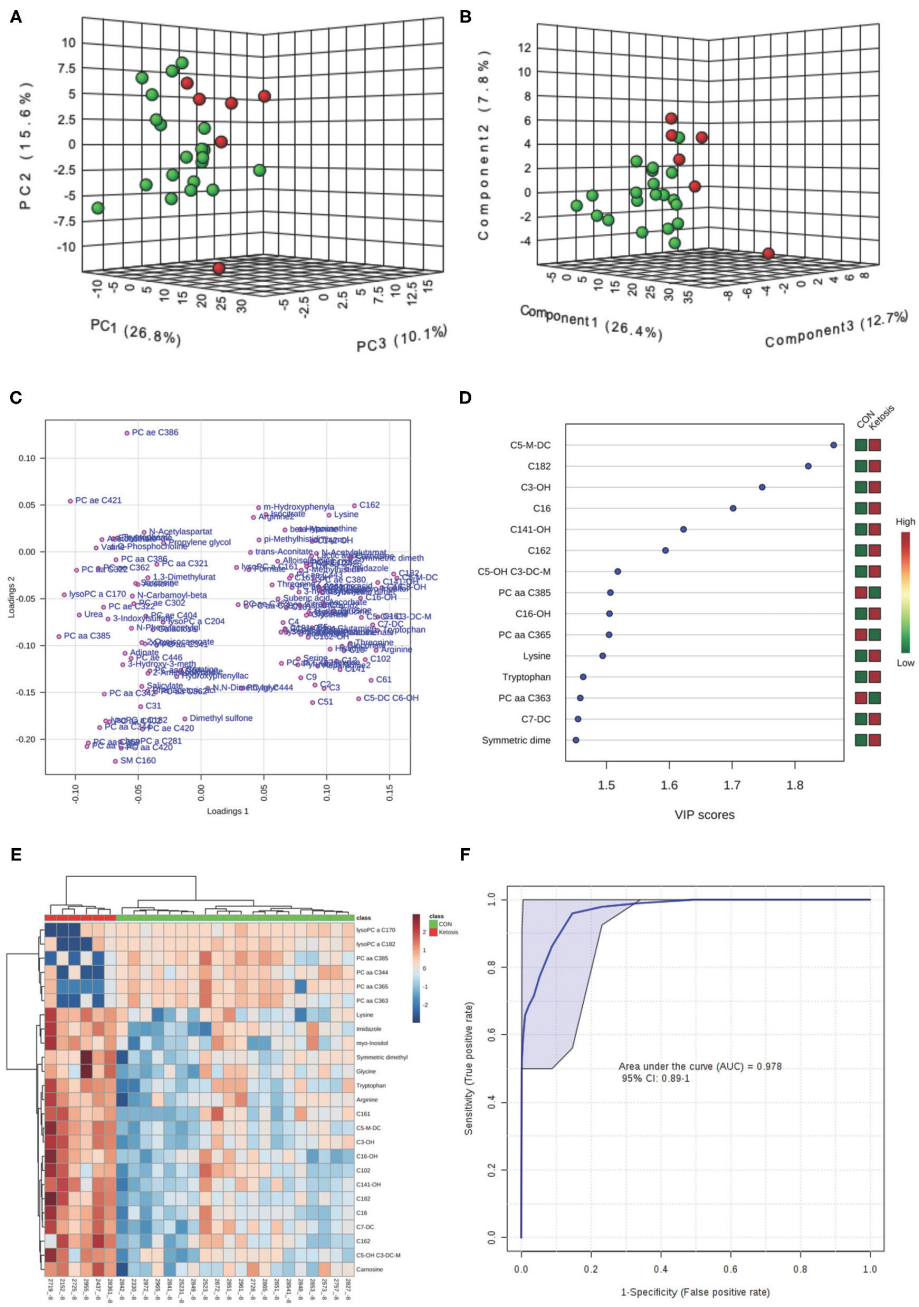
**(A)** Principal component analysis (PCA) and **(B)** Partial least squares-discriminant analysis (PLS-DA, Permutation test: *P* < 0.05) of 20 control (CON, Green) and 6 pre-ketotic (i.e., ketosis group; Red) cows at −8 weeks before parturition showing 2 separated clusters for 2 groups; **(C)** Loading plot for PLS-DA model; **(D)** Variables ranked by variable importance in projection (VIP); **(E)** Heat map based on PLS-DA VIP scores and top 25 metabolites/lipids; and **(F)** Receiver-operator characteristic (ROC) curve of 20 CON and 6 pre-ketotic (i.e., ketosis group) cows at −8 weeks before parturition for the top 6 urine variables (i.e., C5-M-DC, C18:2, C3-OH, C16, C14:1-OH, and C16:2; empirical *P* = 0.001).

**Figure 2:**
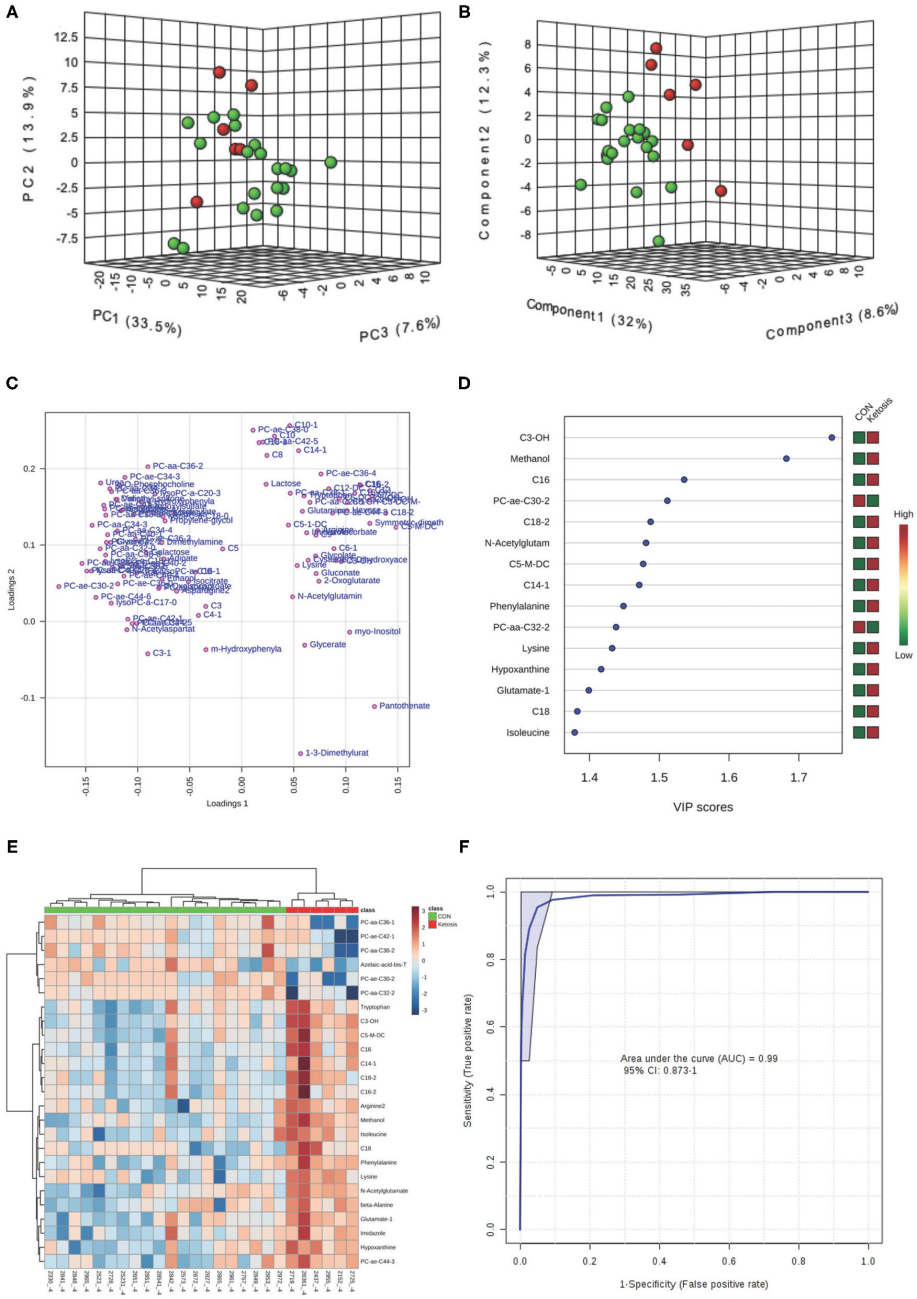
**(A)** PCA and **(B)** PLS-DA (Permutation test: *P* < 0.05) of 20 CON (Green) and 6 pre-ketotic (i.e., ketosis group; Red) cows at −4 weeks before parturition showing 2 separated clusters for 2 groups; **(C)** Loading plot for PLS-DA model; **(D)** VIP; **(E)** Heat map based on PLS-DA VIP scores and top 25 metabolites/lipids; and **(F)** ROC curve of 20 CON and 6 pre-ketotic (i.e., ketosis group) cows at −4 weeks before parturition for the top 4 urine variables (i.e., C3-OH, methanol, C16, and PC ae C30:2; empirical *P* = 0.003).

In the multivariate analysis, both 3-dimensional principal component analysis (PCA) and partial least squares—discriminant analysis (PLS-DA) score plots revealed clearly distinguished clusters between the two groups of cows based on a combination of all urinary metabolites/lipids measured from three metabolic profiling instruments at −8 and −4 weeks (Figures 1A,B, 2A,B). The data from −8 weeks showed that metabolite fingerprints of pre-ketotic cows were separate from those of CON group with principle component (PC) 1 at 26.8%, PC2 at 15.1%, and PC3 at 10.1% for PCA, and PC1 at 26.4%, PC2 at 7.8%, and PC3 at 12.7 for PLS-DA, respectively (Figures 1A,B). To understand the contribution of individual urine metabolite for the variation of the first three PC's (i.e., PC1, PC2, and PC3), we used the corresponding loading plot for both PCA and PLS-DA models.

Examples of loading plots for the PLS-DA models at −8 and −4 weeks are presented in Figures 1C, 2C. The loading plots show similar or distinct behaviors between variables. Metabolites with the same distance and similar directions from 0 are positively correlated, whereas those in opposite directions are negatively correlated. For instance, C5-M-DC, C18:2, C3-OH, C16, and hydroxytetradecenoyl-L-carnitine (C14:1-OH) were positively correlated with each other in pre-ketotic cows. These lipids, however, are negatively correlated with PC aa C38:5 and urea. Moreover, those five urinary lipids that are positively associated with each other in pre-ketotic cows appear to contribute significantly in the separation along the PC1 axis. The *P*-value for a permutation test with 2,000 resampling steps for the PLS-DA model was lower than 0.05.

The variable importance in projection (VIP) plot from PLS-DA ranked the top 15 significant metabolites that contributed mostly to the separation of pre-ketotic cows from CON cows at −8 and −4 weeks prepartum (Figures 1D, 2D). The VIP plots indicated that C5-M-DC, C18:2, C3-OH, C16, C14:1-OH, and hexadecadienyl-L-carnitine (C16:2) at −8 weeks, and C3-OH, methanol, C16, and PC ae C30:2 at −4 weeks were the strongest differential metabolites/lipids in the urine for separating pre-ketotic cows from CON ones. To visualize the relationship and distinction in the levels of urinary metabolites among each sample at −8 and −4 weeks, a heat map was constructed based on the PLS-DA VIP scores and the top 25 metabolites are shown in Figures 1E, 2E. Figure 1E shows that among the top 25 metabolites, concentrations of 19 urinary metabolites (e.g., Lys and myo-inositol) were greater in 6 pre-ketotic cows, whereas the other 6, all of which were lysoPC's (e.g., lysoPC a C17:0) and PC (e.g., PC aa C38:5) groups were lower in pre-ketotic cows at −8 weeks. A 6-lipid biomarker panel (i.e., C5-M-DC, C18:2, C3-OH, C16, C14:1-OH, and C16:2) at −8 weeks, and a 4-metabolite/lipid biomarker panel (i.e., C3-OH, methanol, C16, and PC ae C30:2) at −4 weeks prepartum were built as predictive urine biomarkers for ketosis based on VIP scores. The performance of these biomarkers was evaluated by a receiver operating characteristic (ROC) curve at −8 and −4 weeks using a PLS-DA model (Figures 1F, 2F). The AUC for two ROC curves are 0.978 (95% CI, 0.89-1) with empirical *P*-value = 0.001 (under 1,000 permutations) at −8 weeks and 0.99 (95% CI, 0.873-1) with empirical *P*-value = 0.003 (under 1,000 permutations) at −4 weeks, respectively, which suggests that the urinary biomarkers identified are very strongly predictive for ketosis in dairy cows.

### Urinary Metabolite Alterations During the Week of Ketosis

Results of the univariate analysis during the week of ketosis diagnosis showed that 48 urine metabolites were different (*P* < 0.05) between the two groups of cows (Table 1). The majority of elevated metabolites in the urine of ketotic cows at the disease week were from ACs [e.g., C5-M-DC, dodecanoyl-L-carnitine (C12), and C18:2], and organic acids (i.e., ascorbic acid, glycolic acid, and L-lactic acid) (Table 1). Moreover, concentrations of two urinary ketones [i.e., 3-hydroxybutyric acid (3HBA or BHBA), AcAc] were significantly greater whereas those of acetone (Ac) had a tendency (*P* = 0.07) to be greater in ketotic cows vs. the CON cows (Table 1). On the contrary, concentrations of most PC lipids (e.g., PC ae C30:2, PC aa C40:4, and PC aa C38:3) decreased in cows that developed ketosis during the disease week.

**Figure 3:**
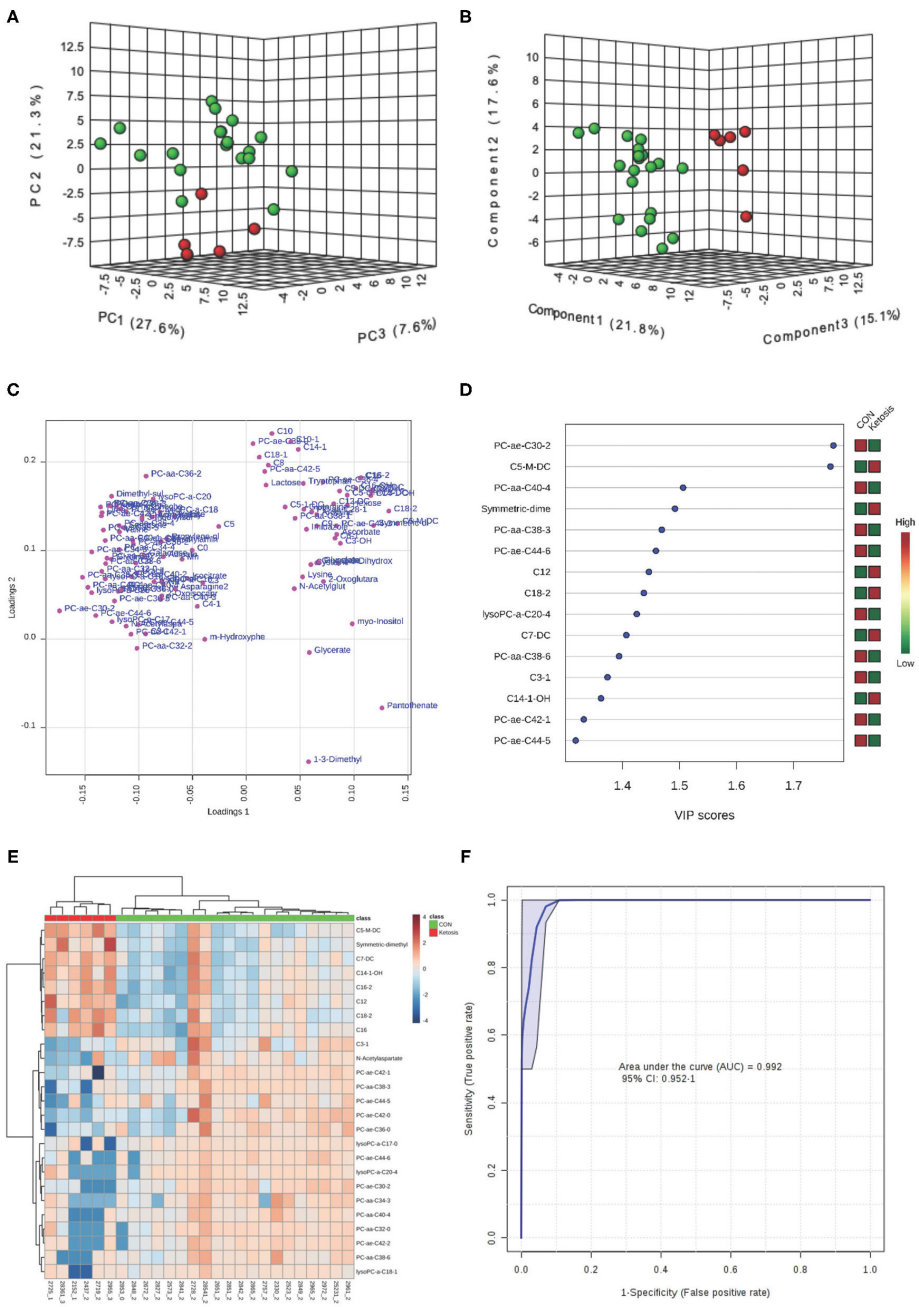
**(A)** PCA and **(B)** PLS-DA (Permutation test: *P* < 0.05) of 20 CON (Green) and 6 ketotic (i.e., ketosis group; Red) cows at disease week showing 2 separated clusters for 2 groups; **(C)** Loading plot for PLS-DA model; **(D)** VIP; **(E)** Heat map based on PLS-DA VIP scores and top 25 metabolites/lipids; and **(F)** ROC curve of 20 CON and 6 ketotic (i.e., ketosis group) cows at disease week for the top 4 urine variables (i.e., PC ae C30:2, C5-M-DC, PC aa C40:4, and SDMA; empirical *P* = 0.001).

Both PCA and PLS-DA (*P*-value < 0.05 in a 2,000-permutation test) analyses indicated consistently separated clusters between ketotic and CON cows (Figures 3A,B). Figures 3C,E shows the PLS-DA loading plot and the heat map with the top 25 metabolites based on PLS-DA VIP. Four metabolites including PC ae C30:2, C5-M-DC, PC aa C40:4, and SDMA (Figure 3D) were selected as new diagnostic biomarkers for ketosis. The 4-metabolite/lipid biomarker set was assessed by ROC curve analysis and exhibited very high sensitivity and specificity with an AUC at 0.992 (95% CI: 0.952-1) (Figure 3F). The empirical *P*-value was 0.001 for the 1,000-permutation test of the ROC curve.

### Post-ketosis Urinary Alterations

Comparisons of urinary profiles between the two groups of cows by a *t*-test or Wilcoxon Mann Whitney test revealed that post-ketotic cows experienced alterations in 16 and 31 urine metabolites at +4 and +8 weeks, respectively (Supplementary Tables 2, 4, 6). No differences were observed regarding concentrations of three ketone bodies (BHBA, AcAc, and Ac) in the urine between the two groups, although all of them were numerically greater in post-ketotic cows at +4 and +8 weeks after parturition (Supplementary Table 4). In the absence of ketosis, post-ketotic cows still had 8 and 20 ACs elevated at +4 and +8 weeks after parturition (Supplementary Table 2). Interestingly, numerous PCs were elevated in post-ketotic cows, especially, during the +8 weeks (11 PC's increased in cows with ketosis) (Supplementary Table 2).

Multivariate analyses including PCA and PLS-DA showed clear separated clusters between the two groups of cows at +4 and +8 weeks postpartum (Supplementary Figures 1A,B, 2A,B). The PLS-DA loading plots and heat maps, showing the top 25 metabolites for each sample at two postpartum time points, are shown in Supplementary Figures 1C,E, 2C,E. The VIP plots indicate that the top four urine metabolites (i.e., PC ae C36:4, C16:2, *N*-acetylglutamic acid, and C5-M-DC) at +4 weeks, and the top five urine [i.e., decenoyl-L-carnitine (C10:1), methylmalonyl-L-carnitine/hydroxyvaleryl-L-carnitine: C5-OH (C3-DC-M), PC aa C38:1, C16:2, and C3-OH] at +8 weeks were significantly different between post-ketotic cows and CON cows (Supplementary Figures 1D, 2D). ROC curves for the two panels of differential metabolites/lipids revealed that the AUC for the four-metabolite set at +4 weeks and the five-lipid set at +8 weeks were both 1 (95% CI, 1-1) (Supplementary Figures 1F, 2F).

### Metabolic Pathways Associated With the Onset and Progression of Ketosis

Both univariate and multivariate analyses revealed significant alterations of urinary metabolites in the pre-ketotic cows starting at −8 weeks prepartum, ketotic (at diagnosis week) as well as in post-ketotic cows at +8 weeks parturition. Biomarker analysis also identified highly specific predictive and diagnostic biomarkers for ketosis or discriminators between the two groups of cows at different time points. Although some biomarker overlap appeared consistently before and at the week of diagnosis, most biomarkers of the perspective of disease are restricted to a certain sampling time. In order to have a better understanding of ketosis from the disease initiation to progression, and until after recovery, a longitudinal view of metabolic alterations and screening consistently perturbed metabolites at different stages of ketosis is warranted.

Three, all ACs (i.e., C5-M-DC, C16, and C16:2), were elevated at all five tested time points (Table 1 and Supplementary Tables 2, 4, 6). Moreover, another 9 metabolites/lipids including 3 ACs [i.e., C3-OH, C5-OH (C3-DC-M), and pimelyl-L-carnitine (C7-DC)], 3 PC (i.e., PC aa C32:2, PC ae C30:2, and PC ae C44:3), SDMA, pantothenic acid, and myo-inositol before and during the week of diagnosis (i.e., three time points at −8, −4 weeks, and disease week) (Table 1). Interestingly, after the 3 ketone bodies in the urine of ketotic cows returned to similar levels with those of CON cows.

Metabolic pathway analysis such as metabolite sets enrichment analysis was done by MetaboAnalyst 3.0 (20, 21) at five different time points. A few databases including “The Small Molecule Pathway Database” (SMPDB) (22, 23), “The Urine Metabolome Database” (UMD) (19), “The Bovine Metabolome Database” (BMDB) (https://bovinedb.ca/) (24), and “The Kyoto Encyclopedia of Genes and Genomes” (KEGG) (25) were referenced in the summary of altered metabolic pathways during ketosis. Significant altered urine metabolites and corresponding metabolic pathways (the main 50 pathways are listed) at the five tested time points are shown in Table 2. In particular, fatty acid transport metabolism (FFT) and lipid catabolism (LC) were significantly perturbed in cows with ketosis at all 5 time points from −8 to +8 weeks. Besides, glycerophospholipid metabolism (GPL), free fatty acid metabolism (FFAM), methane metabolism (MM), beta-alanine metabolism (BAM), pantothenate and CoA biosynthesis (PCB), galactose metabolism (GAM), and inositol metabolism (IM) were altered before and during diagnosis of ketosis.

**Table 2:** Significant metabolic pathways involved in the onset and progression of ketosis in dairy cows.

**Metabolite**	**Metabolic pathway^f^**
**Fatty acid metabolism**	
C0^e^	FFT, LC
C10:1^a, b, d, e^	FFT, LC
C10:2^a, b, e^	FFT, LC
C12^c, d^	FFT, LC
C12-DC^a, e^	FFT, LC
C12:1^e^	FFT, LC
C14:1^a, b^	FFT, LC
C14:1-OH^a, c, e^	FFT, LC
C14:2-OH^a^	FFT, LC
C16^a, b, c, d, e^	FFT, LC
C16-OH^a, c, d^	FFT, LC
C16:1-OH^e^	FFT, LC
C16:1^a, b^	FFT, LC
C16:2^a, b, c, d, e^	FFT, LC
C16:2-OH^e^	FFT, LC
C18^a, b, d, e^	FFT, LC
C18:1^a, e^	FFT, LC
C18:1-OH^b, e^	FFT, LC
C18:2^b, c, d, e^	FFT, LC
C3-OH^a, b, c, e^	FFT, LC
C3:1^a, c^	FFT, LC
C5^a, e^	FFT, LC
C5-M-DC^a, b, c, d, e^	FFT, LC
C5-OH (C3-DC-M)^a, b, c, e^	FFT, LC
C5-DC (C6-OH)^a, c, e^	FFT, LC
C6:1^a^	FFT, LC
C7-DC^a, b, c^	FFT, LC
C8^a, e^	FFT, LC
**Glycerophospholipid metabolism**	
lysoPC a C18:0^c^	GPL, FFAM, MC
lysoPC a C18:2^b^	GPL, FFAM, MC
lysoPC a C20:3^e^	GPL, FFAM, MC
lysoPC a C20:4^c^	GPL, FFAM, MC
lysoPC a C28:1^a^	GPL, FFAM, MC
PC aa C28:1^a^	GPL, FFAM, MC
PC aa C30:0^e^	GPL, FFAM, MC
PC aa C32:0^b, c^	GPL, FFAM, MC
PC aa C32:2^a, b, c^	GPL, FFAM, MC
PC aa C34:1^b^	GPL, FFAM, MC
PC aa C34:2^a^	GPL, FFAM, MC
PC aa C34:3^c^	GPL, FFAM, MC
PC aa C34:4^a^	GPL, FFAM, MC
PC aa C36:0^a, e^	GPL, FFAM, MC
PC aa C36:3^a^	GPL, FFAM, MC
PC aa C36:4^b, c^	GPL, FFAM, MC
PC aa C36:5^a^	GPL, FFAM, MC
PC aa C38:0^a, e^	GPL, FFAM, MC
PC aa C38:1^e^	GPL, FFAM, MC
PC aa C38:3^b, c^	GPL, FFAM, MC
PC aa C38:5^a^	GPL, FFAM, MC
PC aa C38:6^b^	GPL, FFAM, MC
PC aa C40:1^c, e^	GPL, FFAM, MC
PC aa C40:4^c^	GPL, FFAM, MC
PC aa C40:6^e^	GPL, FFAM, MC
PC aa C42:0^a^	GPL, FFAM, MC
PC aa C42:4^b^	GPL, FFAM, MC
PC aa C42:5^b, c^	GPL, FFAM, MC
PC aa C42:6^a^	GPL, FFAM, MC
PC ae C30:2^a, b, c^	GPL, FFAM, MC
PC ae C36:0^c^	GPL, FFAM, MC
PC ae C36:1^a, d^	GPL, FFAM, MC
PC ae C36:2^a^	GPL, FFAM, MC
PC ae C36:3^a^	GPL, FFAM, MC
PC ae C36:4^a, b, c, d, e^	GPL, FFAM, MC
PC aa C36:5^a^	GPL, FFAM, MC
PC ae C38:0^a, d, e^	GPL, FFAM, MC
PC ae C38:6^b, e^	GPL, FFAM, MC
PC ae C40:3^e^	GPL, FFAM, MC
PC ae C42:0^c^	GPL, FFAM, MC
PC ae C42:1^a, c^	GPL, FFAM, MC
PC ae C42:5^c^	GPL, FFAM, MC
PC ae C44:3^a, b, c, d^	GPL, FFAM, MC
PC ae C44:4^e^	GPL, FFAM, MC
PC ae C44:6^c^	GPL, FFAM, MC
O-Phosphocholine^c^	GPL
**Sphingolipid metabolism**	
SM C16:0^a, b^	MC, CS, SM
**Amino acid metabolism**	
L-Arginine^a, b, e^	APM, UC, GNG, PB
L-Asparagine^e^	AM, AR, GNG, PB
L-Aspartic acid^a, b^	BAM, APM, UC, AM, GNG, PB, MAS
L-Glutamine^a, d^	UC, AR, GM, PPM, GNG, PB
Glycine^a, d^	GNG, GTM, PPM
L-Threonine^a^	GSTM, GNG, PB, KTG, PDA, MM
L-Tryptophan^a, b, e^	GNG, PB, KTG, FM, PDA
Carnosine^a, b, e^	BAM, HM
3-Aminoisobutyric acid^b^	PPM
beta-Alanine^a^	BAM, GNG, AM, PPM, PNM
L-Alloisoleucine^b^	A stereo-isomer of L-Isoleucine
L-Cysteine^b^	GNG, PB, GTM, GSTM, MEM, PCB
L-Isoleucine^b^	GNG, KTG, PB, VLID
L-Lysine^a, b^	KTG, PB, PNM, LD, BM
L-Phenylalanine^b^	GNG, KTG, PB, PTM
L-Tyrosine^a^	GNG, KTG, PB, PTM, CB
L-Valine^c^	GNG, PB, PNM, VLID
N-Acetylaspartic acid^a, c^	ASGM
N-Acetylglutamic acid^a, b, d^	AB, BAB
Pantothenic acid^a, b, c^	BAM, PCB
Tiglylglycine^b^	An acyl glycine
1-Methylhistidine^b^	HM
3-Methylhistidine^a^	BAM, HM
2-Hydroxybutyric acid^b^	PNM
Ascorbic acid^a, c^	GTM
Methylmalonic acid^b, d^	VLID, PNM, PPM
Urea^a, b, c^	APM, UC, PPM
**Ketone body metabolism**	
3-Hydroxybutyric acid^c^	KBM
Acetoacetic acid^c^	KBM, PTM, VLID, BUM
**Glycolysis/Gluconeogenesis**	
Hexose^a, c^	N/A
1,3-Dihydroxyacetone (DHA)^c, e^	GLM, GL, MM
Arabinose^b^	ASNSM
D-Galactose^c^	NSM, GAM, SM
L-Lactic acid^c^	GNG, GL, PNM, PYM
**The TCA cycle**	
2-Methylglutaric acid^b^	A metabolite of succinic acid
Citric acid^b^	TCA
Isocitric acid^a^	TCA
**Pentose phosphate pathway**	
Gluconic acid^b^	PPP
**Others**	
Adipic acid^a^	CD
Methanol^a^	PNM, MM
3-Indoxyl sulfate^a^	OS
Dimethyl sulfone^c^	SUM
Dimethylamine^c^	MM
ADMA^a, b^	MM
SDMA^a, b, c^	MM
Hypoxanthine^a, b^	PPM
Imidazole^a, b^	N/A
myo-Inositol^a, b, c^	GAM, IM
Trimethylamine^b^	MM
Uracil^c^	PPM, MM, BAM
Suberic acid^e^	FAO
Azelaic acid^b^	PPM

a *Significant metabolic pathways at −8 weeks before parturition*.

b *Significant metabolic pathways at −4 weeks before parturition*.

c *Significant metabolic pathways at the week of diagnosis of disease*.

d *Significant metabolic pathways at +4 weeks after parturition*.

e *Significant metabolic pathways at +8 weeks after parturition*.

f *FFT, fatty acid transport; LC, lipid catabolism; GPL, glycerophospholipid metabolism; FFAM, free fatty acid metabolism; MC, membrane component; CS, cell signaling; SM, sphingolipid metabolism; APM, arginine and proline metabolism; UC, urea cycle; GNG, gluconeogenesis; PB, protein biosynthesis; AM, aspartate metabolism; AR, ammonia recycling; BAM, beta-alanine metabolism; MAS, malate aspartate shuttle; GM, glutamate metabolism; PPM, purine/pyrimidine metabolism; GTM, glutathione metabolism; serine, glycine; GSTM, and threonine metabolism; KTG, ketogenesis; PDA, protein digestion and absorption; MM, methane metabolism; HM, histidine metabolism; PNM, propanoate metabolism; VLID, valine leucine and isoleucine degradation; MEM, methionine metabolism; PCB, pantothenate and coa biosynthesis; LD, lysine degradation; BM, biotin metabolism; PTM, phenylalanine and tyrosine metabolism; CB, catecholamine biosynthesis; AB, arginine biosynthesis; BAB, biosynthesis of antibiotics; ASGM, alanine aspartate and glutamate metabolism; KBM, ketone body metabolism; BM, butyrate metabolism; GLM, glycerolipid metabolism; GL, glycolysis; ASNSM, amino sugar and nucleotide sugar metabolism; NSM, nucleotide sugars metabolism; GAM, galactose metabolism; PYM, pyruvate metabolism; The TCA cycle, citric acid cycle; PPP, pentose phosphate pathway; CD, caprolactam degradation; OS, oxidative stress; SUM, sulfur metabolism; IM, inositol metabolism; MA, mineral absorption; FAO, fatty acid oxidation*.

## Discussion

We hypothesized that metabolomics approaches will identify metabolite panels in the urine of pre-ketotic, ketotic, and post-ketotic cows that can differentiate ketotic from healthy cows. Indeed, results showed that urinary metabolite profiles were altered prior in pre-ketotic cows starting at −8 and −4 weeks prior to calving, when there was no hyperketonemia present yet. Targeted metabolomics, used in this study, identified several predictive and diagnostic urinary biomarker panels of ketosis. The major metabolites and associated metabolic pathways that are involved during the onset and progression of ketosis also are discussed below.

### Acylcarnitines and Fatty Acid Metabolism

Results of this study showed that cows with ketosis had increased concentrations of several ACs in the urine during the dry off and diagnosis of disease weeks, as well as at +4 and +8 weeks postpartum. The largest number of ACs altered was found at −8 and −4 weeks prepartum and during the week of ketosis diagnosis (20, 14, and 12 species of ACs, respectively). There were also 6 and 18 ACs that were altered during +4 and +8 weeks, respectively. Interestingly, there was a mixture of ACs found altered during all time points in the study including short-chain, medium-chain, and long-chain ACs. It should be noted that ACs are fatty acid esters of L-carnitine. Almost all ACs (except C3) were higher in the urine of ketotic cows. Thus, 6 ACs with short-chain fatty acids (SCAC), 5 ACs with medium-chain fatty acids (MCAC), and 9 ACs with long-chain fatty acids (LCAC) were greater at −8 weeks prepartum. Acylcarnitines are classified based on the number of carbon atoms in their fatty acid ester attached to them. Fatty acids with a total carbon atom number from 1 to 6 are usually classified as SCFAs, those of 7 to 12 carbon atoms are defined as MCFAs, and above 12 carbon atoms as LCFAs. Acylcarnitines play a significant role in regulating the homeostasis of glucose and lipid metabolism. While medium-chain fatty acids (MCFA) can enter mitochondria freely, long-chain fatty acids (LCFA) need carnitine to be transported into mitochondria. LCFA act as fuel for many tissues, including skeletal muscle, via β-oxidation (26). Impairment of FA oxidation is associated with accumulation of ACs and their release into circulation and then excreted through urine. Acylcarnitines are critical substrates for both β-oxidation and ketogenesis (27), and metabolic perturbations of ACs might be closely associated with the development of ketosis. It has been reported in humans that a rapid increase of both short- and long-chain ACs in the plasma/serum and urine occurs during fasting and diabetic ketosis (28–30). It has also been shown that hepatic levels of carnitine-palmitoyl-transferase 1 or 2 (CPT-1 and CPT2), long-chain acetul-CoA dehydrogensase (LCAD), 3-hydroxy-3-methylglutaryl-CoA synthase (HMGCS), and acetyl-CoA carboxylase (ACC) were decreased in ketotic cow (31). In addition, in a companion article, we reported that serum concentrations of several medium- (e.g., decanoyl-L-carnitine) and long-chain (e.g., palmitoyl-L-carnitine and stearoyl-L-carnitine) ACs were consistently higher in pre-ketotic, ketotic, and post-ketotic cows (2).

### LysoPC/PC and Glycerophospholipid Metabolism

Phosphatidylcholines (PCs) are essential and the most abundant phospholipids (varying from 60 to 80% in different species) in all mammalian cells and tissues (32, 33). As the main phospholipids in ruminants, PCs are critical for cell membrane structure, free fatty acid metabolism, lipid absorption, cell signaling, and synthesis of lipoproteins (34). In animals, PCs are biosynthesized from choline, which is obtained from the diet through the choline pathway or via the *de novo* biosynthesis through the methylation of phosphatidylethanolamines (PEs), and then catabolized by the enzyme phosphatidylethanolamine *N*-methyltransferase (PEMT) (35). Both exogenous and endogenous choline are converted into PCs, which constitutes ~95% of the total choline pool in most animal tissues (32). In a rodent study with a choline-deficient diet it was shown that limiting PCs precursors lowered PC synthesis (i.e., a 50% decrease in hepatic PC content) and mice treated with the choline-deficient diet developed severe steatohepatitis, steatosis, and liver failure after 3 days (36). Normal concentrations of PC's are required for secretion of very low density lipoprotein (VLDL) from hepatocytes to remove triglycerides (TG) (37). Results from the current study showed that pre-ketotic and ketotic cows had decreased urinary concentrations of 5 PC's including PC aa C32:2, PC ae C30:2, PC aa C32:0, PC aa C36:4, and PC ae C42:1 during the week of ketosis diagnosis and during the prepartum period. A variety of species from the group of PCs such as PC ae C30:2, PC aa C36:4, PC aa C38:3, PC aa C40:4, and PC aa C38:3 that were decreased during the disease week can be used as diagnostic biomarkers of ketosis. Interestingly, alterations in the urine concentration of PCs continued to be present in post-ketotic cows with 4 and 11 PCs being increased at +4 and +8 weeks postpartum, respectively.

Other studies have reported alterations of PCs in the blood of periparturient dairy cows. Thus, a metabolomics study in dairy cows identified several PC biomarkers of hepatic lipidosis (also known as fatty liver, which is a complication of type II ketosis) including PC aa C36:4, PC aa C38:3, PC aa C40:4, and PC aa C38:3, which were lower in the serum of cows with hepatic lipidosis (37). Our results of urine PC profiles in cows with ketosis are in agreement with the serum PC profiles of ketotic cows and cows with hepatic lipidosis (2, 38). In a companion article we reported increased concentrations of 3 PCs at ketosis diagnosis week and 31 elevated PCs at +4 weeks postpartum (2). Lowered concentrations of PC's in the urine of pre-ketotic cows in our study at −8 and −4 weeks prepartum suggests that those cows might have been in their early stages of mild hepatic lipidosis. Human studies have shown that PC alleviates hepatic steatosis. It might be possible that lower urinary PCs in pre-ketotic and ketotic cows might suggest utilization of PCs by the host potentially for easing fatty liver. Since pre-ketotic cows and CON cows had no difference in the dry matter intake (DMI) at −8 and −4 weeks prepartum, perturbations of the choline pathway might not be the main reason for shortage of PC biosynthesis. It was reported that the PEMT pathway for PC biosynthesis, catalyzed by the transferase enzyme PEMT, is uniquely significant in the liver (39). Therefore, the second pathway (i.e., the endogenous pathway for PC biosynthesis in animals) by PEMT also might be suppressed in cows with ketosis, which lowers secretion of VLDL and further contributes to the accumulation of TG in the liver affecting liver functions.

### Amino Acid Metabolism

Besides their role as building blocks of polypeptides and proteins, AAs also are important precursors for gluconeogenesis and ketogenesis (40, 41), and critical regulators or intermediates in various metabolic pathways that are associated with maintenance, growth, reproduction, immunity, cell signaling, and oxidative stress (42–44). Therefore, homeostasis of amino acid metabolism is vital for maintaining health and preventing metabolic or infectious diseases. In this study, we found that several AAs [e.g., L-arginine (L-Arg), L-aspartic acid (L-Asp), L-glutamine (L-Gln), glycine (Gly), β-alanine (β-Ala), L-cysteine (L-Cys), L-isoleucine (L-Ile), L-lysine (L-Lys), L-phenylalanine (L-Phe), and L-tyrosine (L-Tyr)] and their metabolic products such as ADMA, SDMA, carnosine, N-acetylglutamic acid, pantothenic acid, 1-methylhistidine, and 3-methylhistidine] were increased in the urine of pre-ketotic cows at one or more of the time points, especially at −8 and −4 weeks prior to parturition. At +4 and +8 weeks postpartum concentrations of L-Gln, Glyc, and L-Arg in the urine of post-ketotic cows had no specific trend. For example, L-Gln and Gly were lower at +4 weeks and L-Arg was not affected. However, at +8 weeks postpartum L-Gln and L-Arg were higher in the urine of post-ketotic cows whereas Gly was lower.

A disturbance of AA metabolism in ketotic cows has been previously noted (2, 4). In the current study, concentration of L-Arg in the urine was greater, whereas that of urea was lower in ketotic cows. In fact, the amount of urea in the urine of pre-ketotic (at −8 and −4 weeks prepartum) and ketotic cows (ketosis diagnosis week) were 3.51-, 3.24-, and 4.45-fold lower, respectively, compared with CON cows. A comparative proteomic study of liver tissue showed that arginase-1, a key enzyme that catalyzes the first step of Arg degradation through the urea cycle, was decreased in cows with ketosis (45). Our data are consistent with the reported finding of Xu and Wang (45) that showed that degradation of Arg is suppressed due to the downregulation of hepatic arginase-1 in ketotic cows. Our data also fit with a previous report by Kayano and Kataoka (46) who showed that during ketosis milk urea was lower in those cows. Interestingly, ADMA and SDMA, both analogs of L-Arg, were increased in the urine of our pre-ketotic cows. The ratio of ADMA to SDMA and L-Arg to ADMA in the plasma and urine have been used to indicate renal disease and inflammatory states. The ratio of ADMA to SDMA in the urine of cows with ketosis and CON cows were 0.46-, 0.21-, 0.08-, 0.07-, and 0.19-fold and 0.79-, 0.62-, 0.31-, 0.28-, and 0.44, respectively, at −8, −4, ketosis diagnosis week, and at +4 and +8 weeks postpartum. It is apparent that the amount of SDMA increases in pre-ketotic and post-ketotic cows. Asymmetric and symmetric dimethylarginine derive from hydrolysis of methylated arginines present in various proteins. SDMA and ADMA play significant roles in the pathophysiology of multiple diseases because they are involved in inhibition of NO synthase, an enzyme involved in the production of NO and also by inhibiting the cellular uptake of L-arginine, a NO precursor. Emerging clinical and experimental evidence indicates that ADMA and SDMA are involved in oxidative stress (47, 48), inflammation (49, 50), apoptosis (51), and impaired immunological functions (52). SDMA has been used as a diagnostic marker of kidney function, glomerular filtration rate, and as an inhibitor of NO synthesis (53). The ratio of L-Arg to ADMA in the urine also increased in both ketotic and CON cows as follows: 0.70-, 0.66-, 1.94-, 2.84-, and 1.03-fold in ketotic cows and 0.46-, 0.51-, 1.46-, 1.81, and 1.13-fold in CON cows at −8, −4, ketosis diagnosis week, +4 and +8 weeks around parturition. In human medicine the ratio of L-Arg to ADMA has been identified as an independent risk factor for multiple diseases and mortality in critically ill patients (54). To our best knowledge this is the first time that the ratio between ADMA/SDMA and L-Arg/ADMA have been suggested in dairy cows to point out differences between ketotic and healthy cows.

Degradation of amino acids in the muscle tissue is an important way to produce glucose through the process of gluconeogenesis when the host is in need of glucose like cows going through ketosis. Previously we reported that the sum of hexoses (95% glucose) in the serum of pre-ketotic cows was elevated compared to healthy cows (11). Catabolism of amino acids is associated with the release of large amounts of ammonia (${\text{NH}}_{3}^{+}$) ions which need to be removed from the body. Urea is one of the major nitrogenous excretory products, very important for disposal of ammonia, a highly toxic compound. Several amino acids including Arg, Asp, Gln, and Gly contribute to the urea cycle and serve also as ammonia carriers and excretory products of ammonia in the urine (55). Therefore, one potential reason for excretion of the aforementioned amino acids in the urine of pre-ketotic and ketotic cows (less in post-ketotic cows) might be related to the fact that urea synthesis in the liver is decreased and therefore excretion of ammonia is realized through excretion of those amino acids that transport ammonia out of the body.

Besides being a carrier for ${\text{NH}}_{3}^{+}$, urea is important in acid-base regulation. Pre-ketotic cows in our study were in a state of lactic acidosis prepartum and then in a state of ketoacidosis postpartum. In a companion article (11) we reported greater lactic acid in the serum of pre-ketotic (−8 and −4 weeks prepartum) cows and higher BHBA in ketotic cows (ketoacidosis). Synthesis of urea requires HCO${\text{HCO}}_{3}^{-}$ ions (56). Given that pre-ketotic cows were in a state of metabolic acidosis, removal of bicarbonate ions through urea synthesis in the liver is inhibited to save bicarbonate ions important to regulate acidic-base balance in the body.

Besides their roles in gluconeogenesis or ketogenesis AAs also play significant roles in immune functions (43). Indeed, in a companion article we reported that pre-ketotic cows had greater concentrations of interleukin-6 (IL-6), tumor necrosis factor (TNF), and haptoglobin in the serum starting at −8 and −4 weeks before parturition vs. CON cows (11). Elevated concentrations of these innate immunity reactants in the serum of pre-ketotic cows during the dry-off period indicates that an acute phase response or systemic immune response was present. An unidentified factor during the dry-off period might be involved in activation of innate immune responses during this time period promoting excessive breakdown of proteins from muscles to release sufficient AAs, necessary for biosynthesis of antimicrobials like antibodies, cytokines, and APP. Moreover, it has been reported that 6 functional AAs including Arg, Cys, Gln, Leu, Pro, and Trp play important roles in enhancing immune status of the body and regulating lymphocytes and macrophages' response to bacterial antigens (43).

Six metabolites including β-Ala, carnosine, L-Asp, pantothenic acid, and 3-methylhistidine, which are involved in beta-alanine metabolism were elevated in the urine of pre-ketotic cows at −8 and −4 weeks prepartum. Carnosine (mostly found in muscle and brain tissues) is a dipeptide made of the precursors β-Ala and L-histidine (L-His) and has been reported to be an important antioxidant scavenging reactive oxygen species in different pathologies (57, 58). Several *in vivo* studies have demonstrated that carnosine and His can lower hepatic TG and cholesterol in diabetic condition and alleviate hepatic steatosis (59, 60). The reason for their excretion is not clear presently and warrants further research.

Pantothenic acid or vitamin B5, has considerable metabolic importance in production of energy from carbohydrates, fatty acids, and AAs as it is an integral part of CoA, phosphopantetheine, and acyl-carrier-protein (ACP), all of which are involved in fatty acid metabolism (61). Ruminants have two sources of pantothenic acid including feedstuff and biosynthesis by microorganisms in the rumen (62). Increased levels of carnitine, and β-Ala, and a tendency toward increased L-His in pre-ketotic cows suggest that the animal's antioxidant protection response was activated. The reason for elevated levels of pantothenic acid in the urine of cows with ketosis is not known; however, this should raise concerns regarding dairy cow nutrition and emphasizes the need to balance the diet so that the amount of the pantotheic acid excreted is equal to its intake. Increased urinary pantothenic acid in pre-ketotic and ketotic cows suggests that more pantothenic acid is present in the free form rather than conjugated to CoA, fatty acid synthetase, phosphopantetheine, or ACP, which might affect AA or fatty acid metabolism.

### Ketone Body Metabolism and Glycolysis/Gluconeogenesis

Ketosis or hyperketonemia has been traditionally defined as a condition of abnormally increased concentrations of ketone bodies in the blood, urine, and milk of dairy cows. Elimination of ketone bodies in the urine goes contrary to the well-established belief that ketone bodies are produced as alternate energy molecules for a host that lacks glucose. A challenging question can be raised: why dairy cows excrete in the urine and milk ketone bodies that are necessary for the host? Current belief is that the disturbed homeostasis of energy balance in ketotic cows enhances gluconeogenesis, lipolysis, proteolysis, and glycogenolysis, all of which are aimed at providing sufficient energy substrates. Three ketone bodies (i.e., BHBA, AcAc, and Ac) are mostly formed in the liver from fatty acid β-oxidation and also partly from two ketogenic AAs (i.e., Lys and Leu) and 5 ketogenic and glucogenic AAs (i.e., Ile, Phe, Thr, Trp, and Tyr). Urine concentrations of BHBA and AcAc were greater in ketotic cows in this study compared with CON cows during the week of diagnosis. The data regarding urine ketone bodies are consistent with the serum data from the same cows published byb our lab, in a companion article (11). No differences among the three ketone bodies in the urine were observed at other time points in the study between the two groups of cows. Our results indicate that urine ketones are reliable for the diagnosis of ketosis. However, the data suggest that they could not serve as good biomarkers for prediction or monitoring of the risk of ketosis because they increase only during the week of diagnosis of disease and not during prepartum period (at −8 or −4 weeks prepartum).

There were no distinct differences in terms of intermediates or their precursors involved in glycolysis, gluconeogenesis, the TCA cycle, or other energy-related metabolites in the urine of cows with ketosis vs. CON. In particular, concentrations of glucogenic AAs in the urine were not different between the two groups of cows. On the other hand, very different results were obtained from the serum samples showing elevated levels of both glucogenic and ketogenic AAs in ketotic cows (2). This suggests that urinary glucogenic AAs cannot be used as indicators of energy status in cows with ketosis.

Urine concentrations of hexose (i.e., aldohexoses such as D-glucose and D-mannose; and ketohexoses such as D-fructose), 1,3-dihydroxyacetone (DHA), and L-lactic acid were greater, whereas D-galactose was lower in cows with ketosis during the disease week. No differences in the urinary concentration of hexose and L-lactatic acid were identified at +4 and +8 weeks postpartum between the post-ketotic cows and CON ones. Greater levels of L-lactic acid and hexose (mainly glucose) in the urine of ketotic cows agrees with our serum findings (2, 11). However, it is surprising that hexose (mostly glucose) is excreted in the urine of ketotic cows that are in need of glucose. In ruminants, the major precursors for gluconeogenesis are glucogenic volatile fatty acids including propionate, isobutyrate, and valerate as well as L-lactic acid, glycerol, and glucogenic AAs (63). The energy demand for milk production increases immediately after parturition when the mammary gland requires excessive glucose and AAs for the synthesis of milk lactose and protein. As a result the nutritional needs for milk production increases from virtually zero to around 1 kg/day within a few days after parturition (63, 64). A review paper on precursors for liver gluconeogenesis in periparturient dairy cows points out that the most important adaptation of metabolism to meet the increased requirements for glucose in the immediate postpartum period is endogenous recycling of glucogenic carbon originating from the lactic acid (Cori) cycle (63). Increased concentrations of monosaccharides such as hexose and DHA, and precursors of gluconeogenesis (e.g., L-lactic acid) in the urine suggest obstruction of tubular reabsorption of these metabolites in the kidney, producing marked glucosuria.

### Other Metabolites and Related Metabolic Pathways

It is interesting that concentrations of *myo*-inositol in the urine were greater in both pre-ketotic (i.e., at −8 and −4 weeks prepartum) and ketotic (i.e., at the disease week) cows compared with CON ones. No alteration regarding myo-inositol were observed in post-ketotic cows at both +4 and +8 weeks postpartum. *Myo*-inositol is the most abundant form of the 9 stereoisomers of inositol. *Myo*-inositol is an important lipotropic factor and its role in signal transmission for various growth factors, neurotransmitters, and hormones has been well-documented (65). Additionally, it was reported that *myo*-inositol is involved in lipolysis and in lowering blood cholesterol levels (66). *Myo*-inositol deficiency has been recognized as an important factor that might contribute to the accumulation of TG in the liver or intestines. Recently, it was reported that an increased excretion of *myo*-inositol in the urine of human subjects with hyperglycemia suggested *myo*-inositol in the urine is a good marker of glucose intolerance (67). In animals, the initial committed step in the metabolism of *myo*-inositol occurs exclusively in the kidney for the production of D-glucuronic acid, yielding D-xylulose 5-phosphate that enters the pentose phosphate pathway for energy production (68). Additionally, Dona et al. (69) reported that *myo*-inositol administration can lower oxidative abnormalities in patients with polycystic ovary syndrome, suggesting that *myo*-inositol could act as an antioxidant compound. Increased levels of *myo*-inositol in the urine of pre-ketotic and ketotic cows suggest that increased fat mobilization may occur before and during the occurrence of ketosis, which requires more *myo*-inositol to maintain fatty acid metabolism.

It should be noted that we identified several metabolite panels that can be used in the future to screen cows for identifying those that are at risk of developing ketosis or for more accurate diagnosis of ketosis after calving. All cow-side ketone tests used currently by dairy industry lack high accuracy. Lower accuracy might be associated with medication of the wrong cows (false positives) that do not need to be treated or failure to medicate cows that are in need to be treated (false negatives). Therefore, using new and more accurate tests for ketosis is a very desirable outcome for the dairy industry. The higher accuracy (99% sensitivity and specificity) of metabolite panels that we have identified might fill this gap. Finally, we like to caution the readers with regard to our results because of the low number of replicates used. Therefore, our future goal is to validate the identified screening and diagnostic panels of metabolites in a larger cohort of cows.

## Conclusions

Results of this study show that both NMR and MS based metabolomics provides a powerful approach to discover predictive and diagnostic biomarkers of ketosis in dairy cows. A comprehensive analysis of metabolic patterns in the urine before, during, and after the occurrence of ketosis in dairy cows is presented. We identified two panels of early candidate biomarkers of ketosis that have the potential to be used for identifying cows susceptible to ketosis much earlier than the measurement of ketone bodies after parturition. More specifically, a six-lipid biomarker panel (i.e., C5-M-DC, C18:2, C3-OH, C16, C14:1-OH, and C16:2) at −8 weeks, and a 4-metabolite/lipid biomarker set (i.e., C3-OH, methanol, C16, and PC ae C30:2) at −4 weeks, were identified as potential metabolites that discriminate ketotic from healthy cows with excellent sensitivity and specificity. Moreover, four metabolites/lipids including PC ae C30:2, C5-M-DC, PC aa C40:4, and SDMA were selected as biomarkers to characterize ketosis with an AUC at 0.994 (95% CI: 0.969-1). Only a few urinary metabolites were identified to be different between the post-ketotic cows and CON ones. Most of the alterations were identified in pre-ketotic and ketotic cows. Since urine samples are collected non-invasively, the newly identified early predictive and diagnostic urinary biomarkers show remarkable advantage compared to the current “golden standard” of blood biomarkers (e.g., serum BHBA) or urine ketostix test (measurement of AcAc). We realize that the number of cows in this study is low. Therefore, the data should be considered with care and they have to be validated in a larger number of cohorts.

## Data Availability Statement

The original contributions presented in the study are included in the article/Supplementary Material, further inquiries can be directed to the corresponding author.

## Ethics Statement

The animal study was reviewed and approved by The University of Alberta Animal Policy and Welfare Committee.

## Author Contributions

The manuscript was written through contributions of all authors. GZ wrote the first of the draft manuscript, collected samples, maintained the database of the project, did lab analysis, and data statistical analysis. RM contributed in sample analysis and DW contributed in conceiving of the study, supervision of sample analyses, and writing of the manuscript. BA contributed in conceiving the idea and designing of the experiments, supervised the study, lab analyses, statistical processing as well as writing of the manuscript.

## Conflict of Interest

The authors declare that the research was conducted in the absence of any commercial or financial relationships that could be construed as a potential conflict of interest.

## Supplementary Material

Supplementary Figure 1 **(A)** PCA and **(B)** PLS-DA (Permutation test: *P* < 0.05) of 6 CON (Green) and 6 post-ketotic (i.e., ketosis group; Red) cows at +4 weeks after parturition showing 2 separated clusters for 2 groups; **(C)** Loading plot for PLS-DA model; **(D)** VIP; **(E)** Heat map based on PLS-DA VIP scores and top 25 metabolites/lipids; and **(F)** ROC curve of 6 CON and 6 post-ketotic (i.e., ketosis group) cows at +4 weeks after parturition for the top 4 urine variables (i.e., PC ae C36:4, C16:2, *N*-acetylglutamine, and C5-M-DC; empirical *P* < 0.05).

Supplementary Figure 2 **(A)** PCA and **(B)** PLS-DA (Permutation test: *P* < 0.05) of 6 CON (Green) and 6 post-ketotic (i.e., ketosis group; Red) cows at +8 weeks after parturition showing 2 separated clusters for 2 groups; **(C)** Loading plot for PLS-DA model; **(D)** VIP; **(E)** Heat map based on PLS-DA VIP scores and top 25 metabolites/lipids; and **(F)** ROC curve of 6 CON and 6 post-ketotic (i.e., ketosis group) cows at +8 weeks after parturition for the top 5 urine variables [i.e., C10:1, C5-OH (C3-DC-M), PC aa C38:1, C16:2, and C3-OH, empirical *P* < 0.05].

Supplementary Table 1 Concentrations of non-significant urine metabolites [mean (SD)] in healthy control (CON) and ketotic cows at 3 time points (−8 weeks, −4 weeks, and the week of diagnosis of disease) as determined by DI/LC-MS/MS.

Supplementary Table 2 Concentrations of urine metabolites [mean (SD)] in healthy control (CON) and ketotic cows at +4, and +8 weeks after parturition as determined by DI/LC-MS/MS.

Supplementary Table 3 Concentrations of non-significant urine metabolites [mean (SD)] in healthy control (CON) and ketotic cows at 3 time points (−8 weeks, −4 weeks, and the week of diagnosis of disease) as determined by NMR.

Supplementary Table 4 Concentrations of urine metabolites [mean (SD)] in healthy control (CON) and ketotic cows at +4, and +8 weeks after parturition as determined by NMR.

Supplementary Table 5 Concentrations of non-siginficant urine metabolites [mean (SD)] in healthy control (CON) and ketotic cows at 3 time points (−8 weeks, −4 weeks, and the weeks of diagnosis of disease) as determined by GC-MS.

Supplementary Table 6 Concentrations of urine metabolites [mean (SD)] in healthy control (CON) and ketotic cows at +4, and +8 weeks after parturition as determined by GC-MS.
