# Supplementary figures and images for: A Multi-Platform Metabolomics Approach Identifies Urinary Metabolite Signatures That Differentiate Ketotic From Healthy Dairy Cows
Source: Front Vet Sci. 2021 Jan 26;8:595983. doi: 10.3389/fvets.2021.595983 (PMC7871000; doi:10.3389/fvets.2021.595983)

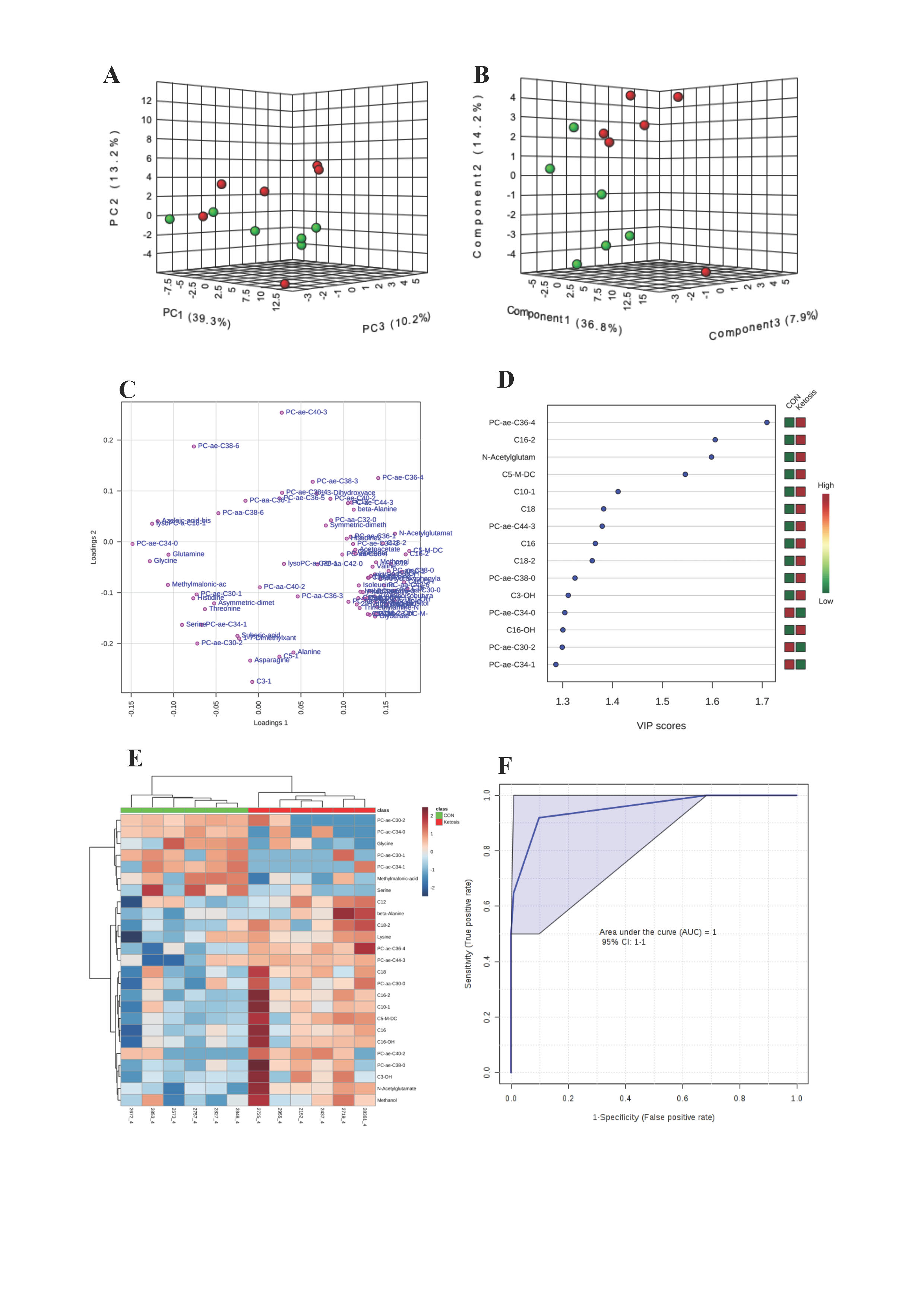

Supplement: Supplementary Figure 1 — (A) PCA and (B) PLS-DA (Permutation test: P < 0.05) of 6 CON (Green) and 6 post-ketotic (i.e., ketosis group; Red) cows at +4 weeks after parturition showing 2 separated clusters for 2 groups; (C) Loading plot for PLS-DA model; (D) VIP; (E) Heat map based on PLS-DA VIP scores and top 25 metabolites/lipids; and (F) ROC curve of 6 CON and 6 post-ketotic (i.e., ketosis group) cows at +4 weeks after parturition for the top 4 urine variables (i.e., PC ae C36:4, C16:2, N-acetylglutamine, and C5-M-DC; empirical P < 0.05). [file Image_1.JPEG]

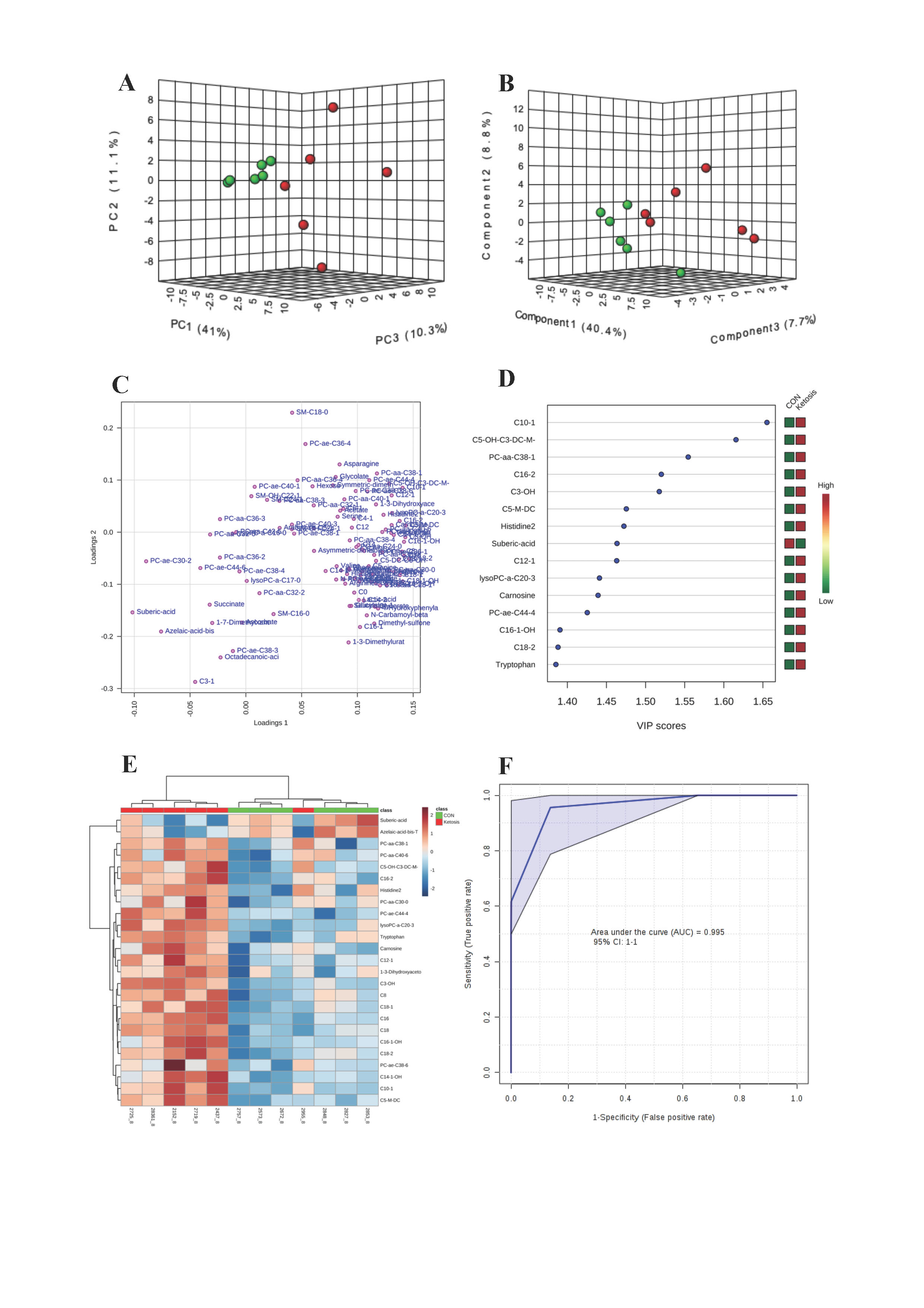

Supplement: Supplementary Figure 2 — (A) PCA and (B) PLS-DA (Permutation test: P < 0.05) of 6 CON (Green) and 6 post-ketotic (i.e., ketosis group; Red) cows at +8 weeks after parturition showing 2 separated clusters for 2 groups; (C) Loading plot for PLS-DA model; (D) VIP; (E) Heat map based on PLS-DA VIP scores and top 25 metabolites/lipids; and (F) ROC curve of 6 CON and 6 post-ketotic (i.e., ketosis group) cows at +8 weeks after parturition for the top 5 urine variables [i.e., C10:1, C5-OH (C3-DC-M), PC aa C38:1, C16:2, and C3-OH, empirical P < 0.05]. [file Image_2.JPEG]
