# Supplementary material for: A Multi-Platform Metabolomics Approach Identifies Urinary Metabolite Signatures That Differentiate Ketotic From Healthy Dairy Cows
Source: Front Vet Sci. 2021 Jan 26;8:595983. doi: 10.3389/fvets.2021.595983 (PMC7871000; doi:10.3389/fvets.2021.595983)
Supplement: Supplementary Table 1 — Concentrations of non-significant urine metabolites [mean (SD)] in healthy control (CON) and ketotic cows at 3 time points (−8 weeks, −4 weeks, and the week of diagnosis of disease) as determined by DI/LC-MS/MS. [file Data_Sheet_1.PDF]

Suppl. Table 1. Concentrations of non-significant urine metabolites (mean (SD)) in healthy control (CON) and ketotic cows at 3 time points (-8 wk, -4 wk, and the wk of diagnosis of disease) as determined by DI/LC-MS/MS

	8 week before parturition					4 week before parturition					Ketosis diagnosis week[1]				
Metabolite, µM/mM creatinine[2]	Ketosis	CON	*P*-value[3]	Fold change	Ketosis / CON	Ketosis	CON	*P*-value	Fold change	Ketosis / CON	Ketosis	CON	*P*-value	Fold change	Ketosis / CON
Number of cases	6	20	-	-	-	6	20	-	-	-	6	20	-	-	
**Acylcarnitines**															
C0	0.914 (0.357)	0.843 (0.267)	0.6002	1.08	Up	1.241 (0.991)	0.863 (0.517)	0.3244 (W)	1.44	Up	0.550 (0.151)	0.848 (0.499)	0.1082 (W)	-1.54	Down
C10	0.053 (0.040)	0.024 (0.009)	0.1365	2.24	Up	0.045 (0.036)	0.027 (0.017)	0.1565 (W)	1.63	Up	0.040 (0.017)	0.030 (0.020)	0.1082 (W)	1.34	Up
C12:1	0.088 (0.110)	0.063 (0.038)	0.6999 (W)	1.4	Up	0.091 (0.125)	0.069 (0.068)	1.0000 (W)	1.31	Up	0.077 (0.039)	0.086 (0.072)	0.8823 (W)	-1.12	Down
C14	0.009 (0.005)	0.007 (0.002)	0.3244 (W)	1.31	Up	0.015 (0.017)	0.007 (0.005)	0.1229 (W)	2.18	Up	0.008 (0.003)	0.007 (0.005)	0.6999 (W)	1.04	Up
C14:2	0.003 (0.001)	0.002 (0.001)	0.6565 (W)	1.09	Up	0.005 (0.004)	0.003 (0.002)	0.2681 (W)	1.81	Up	0.002 (0.001)	0.003 (0.002)	0.7445 (W)	-1.04	Down
C16:1-OH	0.005 (0.003)	0.002 (0.001)	0.0849 (W)	1.9	Up	0.007 (0.007)	0.003 (0.001)	0.4335 (W)	2.2	Up	0.003 (0.001)	0.002 (0.001)	0.3354 (W)	1.21	Up
C16:2-OH	0.007 (0.005)	0.004 (0.001)	0.1409 (W)	1.74	Up	0.012 (0.012)	0.005 (0.002)	0.1101 (W)	2.52	Up	0.005 (0.001)	0.004 (0.001)	0.2027 (W)	1.24	Up
C2	0.121 (0.046)	0.092 (0.038)	0.1301	1.31	Up	0.198 (0.292)	0.111 (0.073)	0.9764 (W)	1.79	Up	0.142 (0.057)	0.124 (0.099)	0.3244 (W)	1.14	Up
C3	0.018 (0.006)	0.013 (0.006)	0.1196	1.33	Up	0.016 (0.006)	0.013 (0.008)	0.1565 (W)	1.26	Up	0.009 (0.006)	0.014 (0.007)	0.2073	-1.46	Down
C3-DC (C4-OH)	0.022 (0.004)	0.020 (0.010)	0.4215 (W)	1.06	Up	0.036 (0.046)	0.021 (0.013)	0.6999 (W)	1.74	Up	0.018 (0.004)	0.025 (0.015)	0.4570 (W)	-1.4	Down
C4	0.107 (0.033)	0.080 (0.046)	0.2129	1.32	Up	0.081 (0.037)	0.080 (0.052)	0.7445 (W)	1.01	Up	0.031 (0.029)	0.030 (0.021)	0.9292 (W)	1.04	Up
C4:1	0.033 (0.024)	0.029 (0.012)	0.6814	1.15	Up	0.041 (0.049)	0.033 (0.015)	0.2681 (W)	1.24	Up	0.023 (0.019)	0.037 (0.025)	0.1756 (W)	-1.55	Down
C6 (C4:1-DC)	0.015 (0.005)	0.012 (0.002)	0.0913 (W)	1.31	Up	0.020 (0.019)	0.017 (0.021)	0.4942 (W)	1.16	Up	0.010 (0.002)	0.010 (0.003)	0.1966 (W)	1.01	Up
C5	0.027 (0.012)	0.019 (0.008)	0.0536 (W)	1.47	Up	0.029 (0.023)	0.022 (0.016)	0.2681 (W)	1.36	Up	0.014 (0.007)	0.017 (0.011)	0.2954 (W)	-1.27	Down
C5:1	0.052 (0.019)	0.042 (0.013)	0.1628	1.23	Up	0.055 (0.057)	0.044 (0.022)	0.5327 (W)	1.24	Up	0.039 (0.011)	0.042 (0.025)	0.8823 (W)	-1.07	Down
C5:1-DC	0.010 (0.010)	0.007 (0.002)	0.9764 (W)	1.47	Up	0.013 (0.016)	0.008 (0.007)	1.0000 (W)	1.56	Up	0.011 (0.002)	0.010 (0.006)	0.2681 (W)	1.09	Up
C9	0.047 (0.017)	0.038 (0.025)	0.1390 (W)	1.24	Up	0.088 (0.096)	0.041 (0.021)	0.2425 (W)	2.13	Up	0.040 (0.017)	0.030 (0.018)	0.2373	1.34	Up
**Lysophosphatidylcholines**															
lysoPC a C16:0	0.019 (0.029)	0.016 (0.011)	0.4335 (W)	1.22	Up	0.022 (0.023)	0.086 (0.203)	0.2218 (W)	-3.86	Down	0.013 (0.008)	0.018 (0.025)	1.0000 (W)	-1.35	Down
lysoPC a C16:1	0.010 (0.007)	0.006 (0.003)	0.1586 (W)	1.55	Up	0.010 (0.019)	0.007 (0.003)	0.0741 (W)	1.53	Up	0.008 (0.012)	0.007 (0.004)	0.1620 (W)	1.17	Up
lysoPC a C17:0	0.003 (0.003)	0.005 (0.002)	0.1583 (W)	-1.75	Down	0.030 (0.019)	0.035 (0.047)	0.4215 (W)	-1.17	Down	0.003 (0.004)	0.004 (0.002)	0.2502 (W)	-1.42	Down
lysoPC a C18:0	0.041 (0.041)	0.032 (0.039)	0.5727 (W)	1.29	Up	0.030 (0.019)	0.035 (0.047)	0.4215 (W)	-1.17	Down	0.015 (0.012)	0.031 (0.031)	0.0949 (W)	-2.02	Down
lysoPC a C18:1	0.003 (0.003)	0.004 (0.003)	0.4332 (W)	-1.46	Down	0.007 (0.010)	0.016 (0.029)	0.1247 (W)	-2.3	Down	0.005 (0.004)	0.007 (0.005)	0.1814 (W)	-1.44	Down
lysoPC a C20:3	0.026 (0.018)	0.017 (0.006)	0.1586 (W)	1.54	Up	0.029 (0.024)	0.019 (0.006)	0.6384 (W)	1.57	Up	0.014 (0.013)	0.019 (0.010)	0.0996 (W)	-1.32	Down
lysoPC a C20:4	0.005 (0.005)	0.003 (0.002)	0.2456 (W)	1.93	Up	0.004 (0.005)	0.006 (0.020)	0.9512 (W)	-1.46	Down	0.004 (0.009)	0.003 (0.004)	0.0571 (W)	1.19	Up
**Phosphatidylcholines**															

PC aa C24:0	0.005 (0.002)	0.005 (0.002)	0.3315 (W)	1.13	Up	0.010 (0.007)	0.005 (0.003)	0.1990 (W)	1.78	Up	0.004 (0.003)	0.005 (0.002)	0.3354 (W)	-1.16	Down
PC aa C30:0	0.027 (0.036)	0.013 (0.005)	0.6838 (W)	2.1	Up	0.013 (0.004)	0.013 (0.008)	0.9750 (W)	-1.05	Down	0.010 (0.006)	0.010 (0.003)	0.4373 (W)	1	Up
PC aa C30:2						0.002 (0.003)	0.001 (0.002)	0.6449 (W)	1.54	Up	0.003 (0.003)	0.001 (0.001)	0.4745 (W)	2.14	Up
PC aa C36:1	0.003 (0.002)	0.018 (0.044)	0.9515 (W)	-5.54	Down	0.003 (0.004)	0.035 (0.110)	0.0940 (W)	-10.78	Down	0.004 (0.004)	0.017 (0.045)	0.6049 (W)	-4.19	Down
PC aa C36:2	0.012 (0.020)	0.058 (0.139)	0.1390 (W)	-4.76	Down	0.006 (0.008)	0.175 (0.633)	0.0634 (W)	-28.4	Down	0.007 (0.005)	0.059 (0.129)	0.2185 (W)	-8.72	Down
PC aa C38:1	0.004 (0.005)	0.003 (0.001)	0.8264 (W)	1.71	Up	0.010 (0.014)	0.004 (0.010)	0.8787 (W)	2.49	Up	0.004 (0.003)	0.002 (0.002)	0.0658 (W)	2.2	Up
PC aa C38:4	0.008 (0.008)	0.022 (0.043)	0.8358 (W)	-2.63	Down	0.024 (0.024)	0.110 (0.433)	0.6565 (W)	-4.63	Down	0.014 (0.009)	0.027 (0.043)	0.7445 (W)	-1.89	Down
PC aa C38:6	0.002 (0.002)	0.017 (0.042)	0.1706 (W)	-8.03	Down	0.003 (0.004)	0.118 (0.490)	0.0552 (W)	-42.28	Down	0.002 (0.002)	0.016 (0.037)	0.1524 (W)	-9.07	Down
PC aa C40:2	0.001 (0.001)	0.002 (0.002)	0.1609 (W)	-2.11	Down	0.003 (0.004)	0.002 (0.005)	0.7840 (W)	1.26	Up	0.001 (0.001)	0.002 (0.002)	0.1204 (W)	-2.07	Down
PC aa C40:3	0.001 (0.002)	0.001 (0.002)	0.7354 (W)	1.04	Up	0.001 (0.002)	0.002 (0.006)	0.4825 (W)	-1.87	Down	0.001 (0.002)	0.001 (0.001)	0.2308 (W)	-1.15	Down
PC aa C40:4						0.002 (0.005)	0.004 (0.012)	0.1346 (W)	-1.8	Down	0.001 (0.001)	0.003 (0.004)	0.0551 (W)	-4.23	Down
PC aa C40:6	0.041 (0.029)	0.026 (0.009)	0.3013 (W)	1.6	Up	0.047 (0.037)	0.060 (0.106)	0.8264 (W)	-1.28	Down	0.019 (0.008)	0.019 (0.012)	0.6411 (W)	-1.02	Down
PC aa C42:1	0.001 (0.001)	0.001 (0.001)	0.8041	1.15	Up	0.002 (0.002)	0.002 (0.002)	0.9292 (W)	-1.1	Down	0.002 (0.003)	0.002 (0.003)	0.8310 (W)	1.26	Up
PC aa C42:2	0.009 (0.005)	0.007 (0.004)	0.2512	1.35	Up	0.008 (0.007)	0.007 (0.006)	0.9292 (W)	1.09	Up	0.007 (0.006)	0.007 (0.005)	0.5327 (W)	-1.02	Down
PC ae C30:0	0.021 (0.020)	0.014 (0.004)	0.7780 (W)	1.49	Up	0.021 (0.017)	0.012 (0.004)	0.5517 (W)	1.69	Up	0.012 (0.010)	0.011 (0.004)	0.4373 (W)	1.09	Up
PC ae C32:2	0.000 (0.001)	0.001 (0.003)	0.0911 (W)	-4.16	Down	0.001 (0.002)	0.002 (0.005)	0.6350 (W)	-1.35	Down	0.002 (0.003)	0.001 (0.003)	0.8297 (W)	1.41	Up
PC ae C34:0	0.001 (0.001)	0.002 (0.002)	0.3452 (W)	-2.23	Down	0.003 (0.002)	0.003 (0.005)	0.6701 (W)	-1.23	Down	0.001 (0.001)	0.002 (0.002)	0.9272 (W)	-1.25	Down
PC ae C34:1	0.001 (0.002)	0.002 (0.001)	0.3012 (W)	-1.21	Down	0.004 (0.004)	0.011 (0.025)	0.2464 (W)	-3.22	Down	0.001 (0.001)	0.002 (0.003)	0.0869 (W)	-2.17	Down
PC ae C34:2											0.002 (0.002)	0.003 (0.006)	0.6914 (W)	-1.57	Down
PC ae C34:3	0.002 (0.003)	0.001 (0.001)	0.7758 (W)	2.54	Up	0.001 (0.002)	0.008 (0.029)	0.5421 (W)	-6.47	Down	0.001 (0.002)	0.001 (0.002)	0.1684 (W)	-1.6	Down
PC ae C36:5	0.001 (0.001)	0.001 (0.001)	0.2331 (W)	-1.35	Down						0.003 (0.006)	0.002 (0.002)	0.1055 (W)	1.61	Up
PC ae C38:1	0.001 (0.001)	0.001 (0.003)	0.4833 (W)	-1.07	Down	0.002 (0.003)	0.003 (0.011)	0.2964 (W)	-1.33	Down	0.002 (0.003)	0.001 (0.002)	0.4424 (W)	1.19	Up
PC ae C38:2	0.001 (0.001)	0.001 (0.001)	0.4711 (W)	1.11	Up	0.001 (0.001)	0.004 (0.012)	0.8789 (W)	-4.7	Down					
PC ae C38:3	0.001 (0.001)	0.001 (0.001)	0.2995 (W)	1.72	Up	0.004 (0.005)	0.007 (0.027)	0.9025 (W)	-2.02	Down	0.002 (0.002)	0.002 (0.002)	0.4745 (W)	-1.06	Down
PC ae C38:4	0.002 (0.001)	0.003 (0.004)	1.0000 (W)	-1.81	Down	0.002 (0.003)	0.016 (0.058)	0.6999 (W)	-6.5	Down	0.001 (0.001)	0.004 (0.005)	0.5427 (W)	-2.81	Down
PC ae C38:5	0.004 (0.006)	0.002 (0.004)	0.9757 (W)	1.75	Up	0.001 (0.001)	0.014 (0.059)	0.4110 (W)	-13.98	Down	0.001 (0.002)	0.003 (0.004)	0.4651 (W)	-1.81	Down
PC ae C40:1	0.002 (0.002)	0.002 (0.001)	0.9251 (W)	1.17	Up	0.003 (0.004)	0.003 (0.010)	0.4105 (W)	1.04	Up	0.001 (0.001)	0.002 (0.002)	0.0666 (W)	-2.42	Down
PC ae C40:2	0.001 (0.001)	0.001 (0.001)	0.4124 (W)	-1.07	Down	0.003 (0.004)	0.006 (0.012)	0.6162 (W)	-1.84	Down					
PC ae C40:3	0.002 (0.004)	0.001 (0.001)	0.5476 (W)	2.85	Up						0.001 (0.002)	0.001 (0.001)	0.3602 (W)	1.32	Up
PC ae C40:4	0.007 (0.004)	0.006 (0.002)	0.0969 (W)	1.09	Up	0.010 (0.011)	0.010 (0.012)	0.5517 (W)	-1.05	Down	0.003 (0.004)	0.006 (0.004)	0.0668 (W)	-1.82	Down
PC ae C40:6	0.003 (0.003)	0.002 (0.001)	0.8753 (W)	1.73	Up	0.002 (0.004)	0.006 (0.022)	0.1201 (W)	-3.35	Down	0.002 (0.002)	0.001 (0.001)	0.6190 (W)	1.29	Up
PC ae C42:2						0.003 (0.003)	0.002 (0.003)	0.3447 (W)	1.65	Up	0.001 (0.001)	0.002 (0.002)	0.0878 (W)	-3.07	Down

PC ae C44:4	0.009 (0.007)	0.006 (0.004)	0.2425 (W)	1.41	Up	0.015 (0.015)	0.008 (0.006)	0.6565 (W)	1.94	Up	0.008 (0.007)	0.008 (0.005)	0.9764 (W)	1.05	Up
PC ae C44:5	0.007 (0.006)	0.007 (0.003)	0.5517 (W)	1.04	Up	0.007 (0.005)	0.008 (0.006)	0.6838 (W)	-1.07	Down	0.004 (0.002)	0.006 (0.002)	0.0874 (W)	-1.64	Down
**Sphingomyelins**															
SM (OH) C16:1	0.001 (0.001)	0.001 (0.000)	1.0000 (W)	1.33	Up	0.004 (0.006)	0.004 (0.015)	0.8021 (W)	-1.02	Down	0.002 (0.004)	0.001 (0.001)	0.6857 (W)	1.93	Up
SM (OH) C24:1	0.002 (0.003)	0.001 (0.001)	0.9512 (W)	3.22	Up	0.001 (0.002)	0.001 (0.003)	0.8247 (W)	1.04	Up	0.000 (0.001)	0.001 (0.001)	0.1881 (W)	-1.56	Down
SM C16:1	0.003 (0.002)	0.003 (0.004)	0.7445 (W)	-1.15	Down	0.002 (0.002)	0.020 (0.080)	0.4825 (W)	-11.03	Down	0.001 (0.001)	0.003 (0.006)	0.7840 (W)	-2.31	Down
SM C18:0	0.003 (0.004)	0.002 (0.001)	0.6827 (W)	1.67	Up	0.005 (0.005)	0.032 (0.104)	0.5025 (W)	-6.8	Down	0.002 (0.003)	0.002 (0.004)	0.7309 (W)	-1.12	Down
SM C22:3						0.001 (0.001)	0.003 (0.007)	0.1487 (W)	-4.28	Down	0.001 (0.000)	0.001 (0.001)	0.6630 (W)	1.02	Up
SM C24:0	0.019 (0.018)	0.009 (0.006)	0.0969 (W)	2.1	Up	0.024 (0.022)	0.033 (0.066)	0.5944 (W)	-1.41	Down	0.008 (0.003)	0.008 (0.005)	0.3675 (W)	-1	Down
SM C24:1	0.003 (0.005)	0.005 (0.006)	0.0731 (W)	-2.02	Down						0.001 (0.002)	0.006 (0.010)	0.0755 (W)	-3.76	Down
**Amino acids**															
L-Alanine	19.211 (4.128)	20.807 (18.063)	0.3244 (W)	-1.08	Down	17.192 (14.016)	17.334 (13.315)	1.0000 (W)	-1.01	Down	28.385 (19.726)	39.377 (39.020)	0.4942 (W)	-1.39	Down
L-Asparagine	5.079 (0.706)	4.528 (1.933)	0.3008	1.12	Up	4.970 (2.480)	4.861 (2.700)	0.7897 (W)	1.02	Up	5.929 (2.269)	7.453 (6.640)	1.0000 (W)	-1.26	Down
L-Glutamate	14.801 (7.770)	15.436 (14.160)	0.6140 (W)	-1.04	Down	15.008 (11.611)	13.192 (7.081)	0.9292 (W)	1.14	Up	12.623 (9.047)	15.732 (12.394)	0.4215 (W)	-1.25	Down
L-Histidine	11.366 (5.978)	6.285 (2.877)	0.0931	1.81	Up	12.140 (8.216)	6.871 (4.543)	0.1390 (W)	1.77	Up	11.617 (7.370)	10.474 (8.571)	0.4942 (W)	1.11	Up
L-Serine	18.766 (7.817)	13.916 (7.957)	0.1229 (W)	1.35	Up	17.212 (9.782)	15.162 (9.308)	0.5727 (W)	1.14	Up	24.178 (8.033)	23.788 (15.278)	0.7445 (W)	1.02	Up

[1]Cows were diagnosed with ketosis (n=6) ranging from wk +1 to +3.

[2] C0: DL-Carnitine; C10: Decanoyl-L-carnitine; C12:1: Dodecenoyl-L-carnitine; C14: Tetradecanoyl-L-carnitine; C14:2: Tetradecadienyl-L-carnitine; C16:1-OH: Hydroxyhexadecenoyl-L-carnitine; C16:2-OH: Hydroxyhexadecadienyl-L-carnitine; C2: Acetyl-L-carnitine; C3: Propionyl-L-carnitine; C3-DC (C4-OH): Malonyl-L-carnitine / Hydroxybutyryl-L-carnitine; C4: Butyryl-L-carnitine; C4:1: Butenyl-L-carnitine; C6 (C4:1-DC): Fumaryl-L-carnitine / Hexanoyl-L-carnitine; C5: Valeryl-L-carnitine; C5:1: Tiglyl-L-carnitine; C5:1-DC: Glutaconyl-L-carnitine; lysoPC a: lysophosphatidylcholine acyl; PC aa: phosphatidylcholine diacyl; PC ae: phosphatidylcholine acyl-alkyl; SM (OH): hydroxysphingomyelin; SM: sphingomyelin; ADMA: Asymmetric dimethylarginine; SDMA: Symmetric dimethylarginine.

[3]*p*-value is calculated with t-test as a default, *p*-value with (W) is calculated by the Wilcoxon Mann Whitney test.

Suppl. Table 2. Concentrations of urine metabolites (mean (SD)) in healthy control (CON) and ketotic cows at +4, and +8 wks after parturition as determined by DI/LC-MS/MS

	4 week after parturition					8 week after parturition				
Metabolite[1], µM/mM creatinine	Ketosis	CON	*P*-value[2]	Fold change	Ketosis / CON	Ketosis	CON	*P*-value	Fold change	Ketosis / CON
**Acylcarnitines**										
C0	1.254 (0.820)	1.200 (0.400)	0.5887 (W)	1.05	Up	1.026 (0.354)	0.803 (0.183)	0.2007	1.28	Up
C10	0.052 (0.029)	0.043 (0.020)	0.5462	1.21	Up	0.043 (0.019)	0.024 (0.006)	0.0578	1.8	Up
C10:1	0.093 (0.077)	0.034 (0.019)	0.0087 (W)	2.76	Up	0.062 (0.029)	0.018 (0.002)	0.0022 (W)	3.49	Up
C10:2	0.024 (0.010)	0.016 (0.006)	0.1146	1.5	Up	0.023 (0.010)	0.011 (0.003)	0.0319	2.05	Up
C12	0.101 (0.028)	0.063 (0.026)	0.034	1.6	Up	0.072 (0.041)	0.039 (0.021)	0.1027	1.87	Up
C12-DC	0.039 (0.017)	0.027 (0.012)	0.1796	1.45	Up	0.035 (0.026)	0.018 (0.005)	0.0411 (W)	1.93	Up
C12:1	0.103 (0.038)	0.066 (0.058)	0.2195	1.56	Up	0.064 (0.028)	0.025 (0.008)	0.0164	2.61	Up
C14	0.014 (0.009)	0.008 (0.003)	0.1691	1.75	Up	0.010 (0.006)	0.006 (0.001)	0.1207	1.85	Up
C14:1	0.011 (0.011)	0.006 (0.003)	0.3197	1.8	Up	0.011 (0.011)	0.003 (0.001)	0.1301	3.56	Up
C14:1-OH	0.010 (0.005)	0.006 (0.002)	0.1542	1.68	Up	0.012 (0.007)	0.003 (0.001)	0.043	3.35	Up
C14:2	0.011 (0.015)	0.003 (0.001)	0.6991 (W)	3.28	Up	0.005 (0.004)	0.003 (0.001)	0.2373	1.84	Up
C14:2-OH	0.031 (0.033)	0.012 (0.011)	0.3095 (W)	2.53	Up	0.053 (0.077)	0.005 (0.001)	0.2403 (W)	10.99	Up
C16	0.014 (0.010)	0.005 (0.002)	0.0087 (W)	2.59	Up	0.013 (0.007)	0.003 (0.001)	0.0176	3.74	Up
C16-OH	0.010 (0.005)	0.005 (0.002)	0.0260 (W)	2.08	Up	0.008 (0.005)	0.003 (0.001)	0.0998	2.31	Up
C16:1	0.027 (0.021)	0.017 (0.010)	0.2957	1.62	Up	0.033 (0.032)	0.013 (0.005)	0.1771	2.62	Up
C16:1-OH	0.005 (0.002)	0.003 (0.001)	0.0805	1.52	Up	0.008 (0.005)	0.002 (0.001)	0.0329	3.61	Up
C16:2	0.007 (0.005)	0.003 (0.001)	0.0022 (W)	2.68	Up	0.006 (0.003)	0.002 (0.001)	0.0043 (W)	3.07	Up
C16:2-OH	0.010 (0.006)	0.006 (0.003)	0.1894	1.67	Up	0.014 (0.010)	0.004 (0.002)	0.0260 (W)	3.25	Up
C18	0.005 (0.002)	0.003 (0.001)	0.0338	1.69	Up	0.006 (0.002)	0.002 (0.001)	0.0118	2.98	Up
C18:1	0.007 (0.003)	0.004 (0.002)	0.1128	1.53	Up	0.006 (0.002)	0.003 (0.001)	0.0254	1.9	Up
C18:1-OH	0.011 (0.007)	0.007 (0.004)	0.5887 (W)	1.51	Up	0.013 (0.008)	0.005 (0.002)	0.042	2.84	Up
C18:2	0.009 (0.005)	0.003 (0.002)	0.0329	2.56	Up	0.008 (0.004)	0.002 (0.001)	0.024	3.21	Up
C2	0.213 (0.160)	0.247 (0.086)	0.3095 (W)	-1.16	Down	0.176 (0.087)	0.144 (0.057)	0.4621	1.22	Up
C3	0.030 (0.029)	0.031 (0.012)	0.4848 (W)	-1.05	Down	0.021 (0.010)	0.022 (0.009)	0.7747	-1.08	Down
C3-DC (C4-OH)	0.037 (0.021)	0.042 (0.016)	0.6385	-1.14	Down	0.032 (0.016)	0.030 (0.014)	0.8308	1.06	Up
C3-OH	0.068 (0.055)	0.023 (0.006)	0.099	3.02	Up	0.064 (0.026)	0.019 (0.007)	0.0066	3.43	Up
C3:1	0.027 (0.022)	0.046 (0.018)	0.1444	-1.67	Down	0.018 (0.012)	0.033 (0.014)	0.0649 (W)	-1.83	Down
C4	0.061 (0.077)	0.063 (0.035)	0.3095 (W)	-1.03	Down	0.056 (0.045)	0.049 (0.033)	0.6991 (W)	1.14	Up

C4:1	0.048 (0.027)	0.054 (0.008)	0.6638	-1.11	Down	0.062 (0.040)	0.032 (0.009)	0.1166	1.98	Up
C6 (C4:1-DC)	0.022 (0.018)	0.020 (0.006)	0.5887 (W)	1.12	Up	0.013 (0.004)	0.016 (0.004)	0.327	-1.19	Down
C5	0.032 (0.029)	0.030 (0.012)	0.4848 (W)	1.07	Up	0.029 (0.009)	0.019 (0.010)	0.0411 (W)	1.5	Up
C5-M-DC	0.066 (0.036)	0.023 (0.007)	0.0327	2.87	Up	0.047 (0.020)	0.017 (0.005)	0.014	2.69	Up
C5-OH (C3-DC-M)	0.065 (0.034)	0.045 (0.012)	0.2398	1.43	Up	0.057 (0.014)	0.028 (0.005)	0.003	2.04	Up
C5:1	0.058 (0.038)	0.070 (0.020)	0.2403 (W)	-1.2	Down	0.050 (0.024)	0.038 (0.007)	0.2631	1.33	Up
C5:1-DC	0.019 (0.009)	0.016 (0.005)	0.4821	1.2	Up	0.013 (0.004)	0.011 (0.003)	0.3248	1.2	Up
C5-DC (C6-OH)	0.025 (0.016)	0.022 (0.006)	0.7264	1.12	Up	0.021 (0.005)	0.014 (0.005)	0.0594	1.44	Up
C6:1	0.026 (0.014)	0.020 (0.004)	0.6991 (W)	1.29	Up	0.023 (0.010)	0.015 (0.003)	0.1259	1.51	Up
C7-DC	0.016 (0.010)	0.011 (0.005)	0.2953	1.44	Up	0.013 (0.006)	0.007 (0.002)	0.0829	1.76	Up
C8	0.037 (0.023)	0.024 (0.005)	0.3095 (W)	1.52	Up	0.031 (0.010)	0.016 (0.006)	0.0089	1.93	Up
C9	0.047 (0.023)	0.064 (0.033)	0.3343	-1.35	Down	0.053 (0.030)	0.041 (0.017)	0.374	1.32	Up
**Lysophosphatidylcholines**										
lysoPC a C16:0	0.033 (0.033)	0.011 (0.007)	0.1641	3.04	Up	0.028 (0.029)	0.005 (0.002)	0.1797 (W)	5.62	Up
lysoPC a C16:1	0.018 (0.036)	0.008 (0.008)	1.0000 (W)	2.26	Up	0.009 (0.004)	0.005 (0.005)	0.3095 (W)	1.68	Up
lysoPC a C17:0	0.012 (0.017)	0.006 (0.008)	0.6304 (W)	1.87	Up	0.006 (0.005)	0.003 (0.002)	0.1744	2.22	Up
lysoPC a C18:0	0.044 (0.047)	0.026 (0.008)	0.6991 (W)	1.71	Up	0.023 (0.025)	0.014 (0.004)	0.9372 (W)	1.64	Up
lysoPC a C18:1	0.006 (0.013)	0.008 (0.008)	0.2130 (W)	-1.36	Down	0.007 (0.007)	0.008 (0.006)	1.0000 (W)	-1.18	Down
lysoPC a C18:2	0.013 (0.011)	0.022 (0.017)	0.3087	-1.67	Down	0.009 (0.004)	0.010 (0.011)	0.8182 (W)	-1.2	Down
lysoPC a C20:3	0.034 (0.050)	0.014 (0.012)	0.5887 (W)	2.37	Up	0.027 (0.010)	0.011 (0.004)	0.0043 (W)	2.42	Up
lysoPC a C20:4	0.013 (0.015)	0.005 (0.005)	0.3048	2.32	Up	0.004 (0.006)	0.002 (0.001)	0.8705 (W)	1.57	Up
lysoPC a C28:1	0.012 (0.017)	0.002 (0.002)	0.2215 (W)	5.4	Up					
**Phosphatidylcholines**										
PC aa C24:0	0.010 (0.011)	0.005 (0.003)	0.3095 (W)	2.02	Up	0.011 (0.010)	0.005 (0.002)	0.0649 (W)	2.21	Up
PC aa C28:1	0.019 (0.019)	0.008 (0.004)	0.2403 (W)	2.41	Up	0.016 (0.006)	0.010 (0.004)	0.0723	1.67	Up
PC aa C30:0	0.021 (0.008)	0.013 (0.006)	0.0693	1.67	Up	0.020 (0.009)	0.009 (0.004)	0.0312	2.06	Up
PC aa C30:2	0.002 (0.002)	0.002 (0.001)	0.5206	1.49	Up	0.003 (0.005)	0.000 (0.000)	0.7976 (W)	10.09	Up
PC aa C32:0	0.012 (0.021)	0.001 (0.001)	0.0904 (W)	12.84	Up	0.004 (0.004)	0.001 (0.001)	0.1432	3.09	Up
PC aa C32:1	0.007 (0.012)	0.001 (0.001)	0.6781 (W)	12.71	Up	0.003 (0.002)	0.001 (0.001)	0.0904 (W)	4.62	Up
PC aa C32:2	0.009 (0.018)	0.004 (0.005)	0.9338 (W)	2.21	Up	0.004 (0.002)	0.003 (0.002)	0.279	1.49	Up
PC aa C34:1	0.009 (0.008)	0.005 (0.002)	0.3662	1.65	Up	0.012 (0.019)	0.005 (0.004)	0.6991 (W)	2.46	Up
PC aa C34:2	0.011 (0.011)	0.009 (0.004)	0.5887 (W)	1.2	Up	0.018 (0.024)	0.011 (0.014)	0.9372 (W)	1.62	Up

PC aa C34:3	0.001 (0.002)	0.000 (0.000)	0.6775 (W)	4.42	Up	0.003 (0.005)	0.001 (0.001)	0.8033 (W)	3.92	Up
PC aa C34:4	0.009 (0.008)	0.013 (0.015)	0.5494	-1.5	Down	0.012 (0.023)	0.003 (0.004)	0.8068 (W)	4.18	Up
PC aa C36:0	0.047 (0.039)	0.032 (0.011)	0.8182 (W)	1.48	Up	0.049 (0.017)	0.028 (0.007)	0.0132	1.8	Up
PC aa C36:1	0.002 (0.002)	0.001 (0.001)	0.2818	1.9	Up	0.004 (0.003)	0.001 (0.001)	0.4848 (W)	3.18	Up
PC aa C36:2	0.006 (0.006)	0.004 (0.004)	0.7483 (W)	1.76	Up	0.007 (0.006)	0.004 (0.002)	0.2116	1.95	Up
PC aa C36:3	0.006 (0.004)	0.003 (0.001)	0.0807	2.23	Up	0.004 (0.004)	0.001 (0.001)	0.2678	2.48	Up
PC aa C36:4	0.004 (0.004)	0.004 (0.003)	0.9081	1.07	Up	0.005 (0.004)	0.002 (0.002)	0.0931 (W)	2.46	Up
PC aa C36:5						0.001 (0.001)	0.001 (0.001)	0.1240 (W)	-1.63	Down
PC aa C38:0	0.015 (0.020)	0.006 (0.003)	0.4848 (W)	2.67	Up	0.007 (0.002)	0.002 (0.002)	0.0037	2.99	Up
PC aa C38:1	0.005 (0.007)	0.003 (0.004)	0.5721	1.57	Up	0.007 (0.002)	0.003 (0.001)	0.0002	2.78	Up
PC aa C38:3	0.004 (0.004)	0.003 (0.002)	0.443	1.65	Up	0.005 (0.005)	0.002 (0.001)	0.2524	2.04	Up
PC aa C38:4	0.009 (0.004)	0.005 (0.002)	0.0931 (W)	1.62	Up	0.013 (0.008)	0.005 (0.002)	0.0696	2.72	Up
PC aa C38:5	0.001 (0.001)	0.001 (0.002)	0.6049	-1.44	Down	0.004 (0.004)	0.002 (0.001)	0.3507	1.86	Up
PC aa C38:6	0.004 (0.004)	0.002 (0.001)	0.2631	1.89	Up	0.002 (0.003)	0.005 (0.007)	0.4704 (W)	-2.1	Down
PC aa C40:1	0.047 (0.043)	0.032 (0.013)	0.9372 (W)	1.48	Up	0.040 (0.014)	0.023 (0.009)	0.0315	1.77	Up
PC aa C40:2	0.004 (0.004)	0.002 (0.002)	0.2994	1.84	Up	0.001 (0.002)	0.001 (0.002)	0.6248 (W)	-1.09	Down
PC aa C40:3	0.003 (0.005)	0.002 (0.002)	0.6085	1.53	Up	0.002 (0.002)	0.000 (0.000)	0.1436	7.19	Up
PC aa C40:4	0.002 (0.001)	0.000 (0.000)	0.1044 (W)	7.91	Up	0.001 (0.001)	0.001 (0.001)	0.5497 (W)	-1.2	Down
PC aa C40:6	0.054 (0.048)	0.023 (0.013)	0.17	2.39	Up	0.040 (0.014)	0.018 (0.010)	0.0106	2.22	Up
PC aa C42:0	0.014 (0.017)	0.003 (0.003)	0.1655	4.27	Up	0.005 (0.005)	0.004 (0.005)	0.8555	1.12	Up
PC aa C42:1	0.002 (0.003)	0.001 (0.001)	0.5611 (W)	2.06	Up	0.001 (0.002)	0.000 (0.001)	0.4460 (W)	1.81	Up
PC aa C42:2	0.008 (0.008)	0.008 (0.004)	0.9865	-1.01	Down	0.011 (0.009)	0.007 (0.006)	0.6991 (W)	1.57	Up
PC aa C42:4	0.004 (0.006)	0.000 (0.001)	0.4217 (W)	12.77	Up	0.003 (0.004)	0.001 (0.001)	0.9357 (W)	3	Up
PC aa C42:5	0.001 (0.003)	0.000 (0.001)	0.5497 (W)	3.1	Up	0.006 (0.006)	0.002 (0.001)	0.117	3.78	Up
PC aa C42:6	0.059 (0.048)	0.024 (0.014)	0.1401	2.49	Up	0.035 (0.020)	0.016 (0.007)	0.0611	2.24	Up
PC ae C30:0	0.012 (0.004)	0.012 (0.006)	0.7992	-1.06	Down	0.012 (0.005)	0.010 (0.003)	0.4027	1.22	Up
PC ae C30:2	0.003 (0.007)	0.002 (0.001)	0.0870 (W)	1.4	Up	0.002 (0.003)	0.002 (0.002)	0.1659 (W)	-1.28	Down
PC ae C32:2	0.001 (0.001)	0.001 (0.001)	0.7976 (W)	1.14	Up	0.001 (0.001)	0.000 (0.001)	0.7526 (W)	1.96	Up
PC ae C34:0	0.001 (0.001)	0.001 (0.001)	0.2215 (W)	-1.49	Down	0.002 (0.003)	0.000 (0.001)	0.5320 (W)	4.6	Up
PC ae C34:1	0.001 (0.002)	0.002 (0.002)	0.0874 (W)	-2.58	Down	0.003 (0.006)	0.001 (0.001)	0.8705 (W)	4.69	Up
PC ae C34:2	0.002 (0.004)	0.001 (0.001)	0.9289 (W)	3.02	Up	0.006 (0.013)	0.001 (0.000)	0.5611 (W)	10.44	Up
PC ae C34:3	0.005 (0.006)	0.000 (0.000)	0.1044 (W)	17.57	Up	0.002 (0.002)	0.000 (0.000)	0.1811	6.14	Up

PC ae C36:0	0.010 (0.012)	0.011 (0.006)	0.3939 (W)	-1.19	Down	0.008 (0.004)	0.007 (0.005)	0.4848 (W)	1.1	Up
PC ae C36:1	0.011 (0.006)	0.004 (0.004)	0.0442	2.57	Up	0.009 (0.007)	0.002 (0.001)	0.0698	4.25	Up
PC ae C36:2	0.002 (0.002)	0.002 (0.002)	0.4151 (W)	-1.3	Down	0.004 (0.003)	0.003 (0.001)	0.4263	1.32	Up
PC ae C36:3						0.003 (0.003)	0.002 (0.001)	0.44	1.72	Up
PC ae C36:4	0.014 (0.011)	0.003 (0.002)	0.0022 (W)	4.51	Up	0.018 (0.011)	0.004 (0.004)	0.0256	4.09	Up
PC ae C36:5	0.003 (0.003)	0.001 (0.001)	0.1498	3.23	Up	0.005 (0.011)	0.000 (0.000)	0.6781 (W)	13.33	Up
PC ae C38:0	0.052 (0.057)	0.014 (0.005)	0.0260 (W)	3.61	Up	0.033 (0.011)	0.016 (0.006)	0.0095	2.07	Up
PC ae C38:1	0.003 (0.005)	0.000 (0.001)	0.7750 (W)	9.85	Up	0.002 (0.002)	0.000 (0.001)	0.2805 (W)	4.53	Up
PC ae C38:2	0.001 (0.002)	0.001 (0.001)	0.7973 (W)	1.84	Up	0.001 (0.002)	0.000 (0.000)	0.2458 (W)	4.71	Up
PC ae C38:3	0.003 (0.004)	0.001 (0.001)	0.1999 (W)	5.32	Up	0.002 (0.003)	0.001 (0.001)	0.2807	2.67	Up
PC ae C38:4	0.002 (0.002)	0.001 (0.001)	0.1196	3.39	Up	0.002 (0.002)	0.001 (0.001)	0.1643	2.32	Up
PC ae C38:5	0.001 (0.003)	0.001 (0.000)	0.4550 (W)	2.61	Up	0.001 (0.001)	0.001 (0.001)	1.0000 (W)	1.41	Up
PC ae C38:6	0.011 (0.007)	0.005 (0.003)	0.0764	2.3	Up	0.016 (0.013)	0.006 (0.002)	0.0022 (W)	2.64	Up
PC ae C40:1	0.002 (0.003)	0.002 (0.001)	0.9767	1.02	Up	0.002 (0.001)	0.001 (0.001)	0.1901	2.15	Up
PC ae C40:2	0.007 (0.009)	0.000 (0.001)	0.1147 (W)	14.91	Up	0.001 (0.002)	0.002 (0.004)	0.0874 (W)	-2.27	Down
PC ae C40:3	0.004 (0.006)	0.000 (0.000)	0.0730 (W)	12.74	Up	0.007 (0.005)	0.001 (0.001)	0.0289	9.13	Up
PC ae C40:4	0.006 (0.003)	0.004 (0.004)	0.361	1.43	Up	0.004 (0.004)	0.004 (0.002)	0.7592	1.14	Up
PC ae C40:6	0.002 (0.002)	0.001 (0.002)	0.8033 (W)	1.71	Up	0.002 (0.002)	0.001 (0.001)	0.4069	2.08	Up
PC ae C42:0	0.069 (0.030)	0.059 (0.029)	0.5367	1.18	Up	0.062 (0.029)	0.054 (0.022)	0.6076	1.15	Up
PC ae C42:1	0.005 (0.009)	0.005 (0.003)	0.3751 (W)	-1.01	Down	0.005 (0.004)	0.005 (0.003)	0.7573	-1.13	Down
PC ae C42:2	0.001 (0.001)	0.000 (0.001)	0.7745 (W)	1.66	Up	0.001 (0.002)	0.001 (0.001)	0.5611 (W)	1.14	Up
PC ae C42:5	0.075 (0.063)	0.072 (0.037)	0.6991 (W)	1.04	Up	0.073 (0.037)	0.048 (0.018)	0.1602	1.53	Up
PC ae C44:3	0.008 (0.002)	0.002 (0.002)	0.0050 (W)	4.34	Up	0.006 (0.008)	0.003 (0.002)	0.3213	2.36	Up
PC ae C44:4	0.014 (0.010)	0.008 (0.005)	0.2742	1.68	Up	0.011 (0.005)	0.004 (0.001)	0.0142	2.69	Up
PC ae C44:5	0.006 (0.003)	0.007 (0.006)	0.9372 (W)	-1.22	Down	0.007 (0.006)	0.007 (0.003)	0.8954	-1.05	Down
PC ae C44:6	0.002 (0.002)	0.001 (0.001)	0.6884 (W)	1.31	Up	0.002 (0.002)	0.001 (0.001)	0.2958	1.88	Up
**Sphingomyelins**										
SM (OH) C16:1	0.002 (0.002)	0.001 (0.002)	1.0000 (W)	1.14	Up	0.002 (0.003)	0.000 (0.001)	0.6081 (W)	4.8	Up
SM (OH) C22:1	0.005 (0.009)	0.001 (0.001)	1.0000 (W)	4.25	Up	0.003 (0.004)	0.000 (0.000)	0.2215 (W)	6.33	Up
SM (OH) C24:1	0.000 (0.000)	0.000 (0.001)	0.7745 (W)	-1.37	Down	0.002 (0.002)	0.001 (0.001)	0.1581 (W)	3.58	Up
SM C16:0	0.004 (0.004)	0.003 (0.003)	0.6137	1.41	Up	0.007 (0.009)	0.002 (0.001)	0.2469	3.07	Up
SM C16:1	0.003 (0.004)	0.001 (0.002)	0.3124	2.86	Up	0.002 (0.002)	0.001 (0.001)	0.3221	2.32	Up

SM C18:0	0.000 (0.001)	0.002 (0.001)	0.1140 (W)	-3.48	Down	0.002 (0.001)	0.000 (0.001)	0.0562 (W)	5.52	Up
SM C22:3	0.001 (0.001)	0.000 (0.001)	1.0000 (W)	1.43	Up	0.002 (0.002)	0.000 (0.001)	0.8033 (W)	3.61	Up
SM C24:0	0.024 (0.033)	0.009 (0.005)	0.4848 (W)	2.61	Up	0.009 (0.008)	0.007 (0.003)	0.4481	1.4	Up
SM C24:1	0.001 (0.001)	0.002 (0.002)	0.3403	-1.81	Down	0.001 (0.001)	0.000 (0.000)	0.1224	3.8	Up
Hexose	346.719 (149.641)	340.847 (107.336)	0.9393	1.02	Up	331.868 (139.113)	218.833 (102.644)	0.1403	1.52	Up
**Amino acids**										
L-Alanine	133.817 (218.586)	95.228 (49.189)	0.1320 (W)	1.41	Up	64.069 (36.797)	59.306 (42.245)	0.8392	1.08	Up
L-Arginine	3.947 (1.976)	4.301 (1.924)	0.7596	-1.09	Down	4.720 (1.168)	2.908 (1.206)	0.0260 (W)	1.62	Up
L-Asparagine	8.836 (3.756)	11.126 (3.717)	0.1320 (W)	-1.26	Down	8.725 (2.339)	5.631 (1.976)	0.0328	1.55	Up
L-Aspartic acid	37.396 (53.900)	24.671 (22.303)	0.8182 (W)	1.52	Up	19.896 (3.152)	14.594 (9.936)	0.0649 (W)	1.36	Up
L-Glutamine	48.628 (25.678)	96.033 (48.197)	0.0594	-1.97	Down	64.920 (33.811)	44.943 (29.456)	0.3008	1.44	Up
L-Glutamate	74.605 (109.210)	40.438 (24.117)	0.8182 (W)	1.84	Up	21.532 (12.910)	20.500 (11.842)	0.9372 (W)	1.05	Up
Glycine	156.463 (102.252)	317.795 (141.928)	0.0260 (W)	-2.03	Down	149.078 (140.345)	230.661 (185.570)	0.4105	-1.55	Down
L-Histidine	15.214 (3.045)	23.086 (9.007)	0.0879	-1.52	Down	17.682 (5.664)	12.725 (4.527)	0.125	1.39	Up
L-Serine	27.040 (5.955)	44.854 (18.200)	0.0931 (W)	-1.66	Down	29.967 (9.560)	23.070 (5.752)	0.161	1.3	Up
L-Threonine	16.793 (6.087)	25.282 (8.958)	0.0838	-1.51	Down	20.129 (6.719)	14.938 (10.030)	0.3175	1.35	Up
L-Tryptophan	6.530 (3.163)	5.093 (1.891)	0.3621	1.28	Up	6.873 (2.003)	3.071 (1.542)	0.0042	2.24	Up
**Biogenic amines**										
ADMA	1.386 (0.553)	2.385 (1.596)	0.1964	-1.72	Down	4.570 (3.487)	2.568 (1.644)	0.2323	1.78	Up
SDMA	20.009 (16.649)	8.368 (4.097)	0.1509	2.39	Up	23.187 (22.503)	5.808 (1.339)	0.1172	3.99	Up
Carnosine	3.343 (1.111)	2.866 (1.102)	0.4728	1.17	Up	2.789 (0.814)	1.435 (0.396)	0.0044	1.94	Up

[1] C0: DL-Carnitine; C10: Decanoyl-L-carnitine; C10:1: Decenoyl-L-carnitine; C10:2: Decadienyl-L-carnitine; C12: Dodecanoyl-L-carnitine; C12-DC: Dodecanedioyl-L-carnitine; C12:1: Dodecenoyl-L-carnitine; C14: Tetradecanoyl-L-carnitine; C14:1: Tetradecenoyl-L-carnitine; C14:1-OH: Hydroxytetradecenoyl-L-carnitine; C14:2: Tetradecadienyl-L-carnitine; C14:2-OH: Hydroxytetradecadienyl-L-carnitine; C16: Hexadecanoyl-L-carnitine; C16-OH: Hydroxyhexadecanoyl-L-carnitine; C16:1: Hexadecenoyl-L-carnitine; C16:1-OH: Hydroxyhexadecenoyl-L-carnitine; C16:2: Hexadecadienyl-L-carnitine; C16:2-OH: Hydroxyhexadecadienyl-L-carnitine; C18: Octadecanoyl-L-carnitine; C18:1: Octadecenoyl-L-carnitine; C18:1-OH: Hydroxyoctadecenoyl-L-carnitine; C18:2: Octadecadienyl-L-carnitine; C2: Acetyl-L-carnitine; C3: Propionyl-L-carnitine; C3-DC (C4-OH): Malonyl-L-carnitine / Hydroxybutyryl-L-carnitine; C3-OH: Hydroxypropionyl-L-carnitine; C3:1: Propenyl-L-carnitine; C4: Butyryl-L-carnitine; C4:1: Butenyl-L-carnitine; C6 (C4:1-DC): Fumaryl-L-carnitine / Hexanoyl-L-carnitine; C5: Valeryl-L-carnitine; C5-M-DC: Methylglutaryl-L-carnitine; C5-OH (C3-DC-M): Methylmalonyl-L-carnitine / Hydroxyvaleryl-L-carnitine; C5:1: Tiglyl-L-carnitine; C5:1-DC: Glutaconyl-L-carnitine; C5-DC (C6-OH): Glutaryl-L-carnitine / Hydroxyhexanoyl-L-carnitine; C6:1: Hexenoyl-L-carnitine; C7-DC: Pimelyl-L-carnitine; C8: Octanoyl-L-carnitine; C9: Nonayl-L-carnitine; lysoPC a: lysophosphatidylcholine acyl; PC aa: phosphatidylcholine diacyl; PC ae: phosphatidylcholine acyl-alkyl; SM (OH): hydroxysphingomyelin; SM: sphingomyelin; ADMA: Asymmetric dimethylarginine; SDMA: Symmetric dimethylarginine.

[2] *p*-value is calculated with t-test as a default, *p*-value with (W) is calculated by the Wilcoxon Mann Whitney test.

Suppl. Table 3. Concentrations of non-significant urine metabolites (mean (SD)) in healthy control (CON) and ketotic cows at 3 time points (-8 wk, -4 wk, and the wk of diagnosis of disease) as determined by NMR

	8 week before parturition					4 week before parturition					Ketosis diagnosis week[1]				
Metabolite[2], μM/mM creatinine	Ketosis	CON	*P*-value[3]	Fold change	Ketosis / CON	Ketosis	CON	*P*-value	Fold change	Ketosis / CON	Ketosis	CON	*P*-value	Fold change	Ketosis / CON
Number of cases	6	20	-	-	-	6	20	-	-	-	6	20	-	-	
**Ketones**															
Acetone	8.219 (3.917)	17.460 (21.282)	0.1390 (W)	-2.12	Down	12.164 (8.906)	9.895 (9.997)	0.9292 (W)	1.23	Up	65.841 (41.547)	35.852 (39.485)	0.0726 (W)	1.84	Up
**Saccharides**															
D-Glucose	50.126 (15.552)	42.442 (15.788)	0.3047	1.18	Up	51.996 (26.694)	37.067 (12.683)	0.2356	1.4	Up	55.409 (26.056)	56.344 (33.883)	0.6999 (W)	-1.02	Down
D-Xylose	9.889 (5.206)	11.721 (3.774)	0.1082 (W)	-1.19	Down	11.407 (6.393)	8.092 (2.427)	0.3244 (W)	1.41	Up	9.134 (4.077)	11.860 (5.747)	0.2924	-1.3	Down
Lactose	57.151 (62.335)	30.101 (38.035)	1.0000 (W)	1.9	Up	73.734 (124.581)	36.706 (80.928)	0.1963 (W)	2.01	Up	33.153 (22.719)	24.388 (19.635)	0.2185 (W)	1.36	Up
**Amino acids and derivatives**															
2-Aminobutyric acid	17.163 (11.088)	21.276 (8.442)	0.0828 (W)	-1.24	Down	21.104 (15.266)	15.070 (4.575)	0.9764 (W)	1.4	Up	14.251 (5.555)	19.962 (13.991)	0.4570 (W)	-1.4	Down
Betaine	43.073 (29.269)	41.129 (39.013)	0.4942 (W)	1.05	Up	33.677 (25.259)	20.089 (13.470)	0.2954 (W)	1.68	Up	47.751 (41.546)	44.205 (47.712)	0.8823 (W)	1.08	Up
Creatine	404.335 (231.779)	537.239 (230.096)	0.2273	-1.33	Down	637.932 (241.996)	487.787 (198.752)	0.1349	1.31	Up	963.918 (258.242)	839.356 (327.776)	0.4033	1.15	Up
Guanidoacetic acid	155.972 (252.183)	70.801 (46.911)	0.9764 (W)	2.2	Up	28.911 (11.698)	34.922 (20.700)	0.6999 (W)	-1.21	Down	130.941 (93.853)	86.237 (58.843)	0.3244 (W)	1.52	Up
L-Leucine	335.272 (528.955)	316.259 (310.570)	0.9764 (W)	1.06	Up	90.713 (56.062)	101.260 (131.036)	0.5327 (W)	-1.12	Down	480.603 (183.480)	394.396 (271.802)	0.4763	1.22	Up
L-Methionine	40.199 (66.466)	13.063 (10.452)	0.4215 (W)	3.08	Up	30.351 (48.198)	6.801 (3.568)	0.0949 (W)	4.46	Up	11.069 (9.697)	15.312 (18.697)	0.6565 (W)	-1.38	Down
N,N-Dimethylglycine	16.031 (7.261)	17.210 (5.189)	0.2954 (W)	-1.07	Down	15.690 (5.539)	12.610 (3.410)	0.1069	1.24	Up	18.429 (2.361)	20.130 (9.981)	1.0000 (W)	-1.09	Down
N-Acetylglutamine	14.970 (7.581)	13.584 (4.464)	0.7897 (W)	1.1	Up	13.544 (5.642)	10.846 (2.501)	0.3015	1.25	Up	25.605 (14.652)	19.852 (8.225)	0.2245	1.29	Up
Sarcosine	22.780 (8.574)	35.469 (24.395)	0.1229 (W)	-1.56	Down	19.658 (4.871)	22.229 (9.144)	0.8358 (W)	-1.13	Down	14.866 (7.702)	41.404 (48.702)	0.0720 (W)	-2.79	Down
**Organic acids**															
1,3-Dimethyluric acid	3.171 (1.505)	4.793 (2.528)	0.1565 (W)	-1.51	Down	6.333 (3.785)	3.667 (2.226)	0.1229 (W)	1.73	Up	8.009 (4.550)	8.458 (9.571)	0.2681 (W)	-1.06	Down
2-Hydroxy-3-methylpentanoic acid	49.724 (20.709)	56.393 (39.061)	0.6999 (W)	-1.13	Down	48.550 (13.666)	37.890 (18.706)	0.0720 (W)	1.28	Up	112.934 (56.590)	102.016 (55.232)	0.6764	1.11	Up
2-Hydroxyisobutyric acid	5.858 (1.329)	8.599 (9.199)	0.8358 (W)	-1.47	Down	6.735 (3.393)	4.744 (1.213)	0.1229 (W)	1.42	Up	5.678 (2.543)	5.739 (2.083)	0.9526	-1.01	Down
2-Hydroxyvaleric acid	83.308 (25.381)	87.432 (39.658)	0.6999 (W)	-1.05	Down	73.902 (16.933)	67.675 (19.982)	0.4968	1.09	Up	128.826 (80.920)	104.574 (34.264)	0.5035	1.23	Up
2-Oxobutyric acid	13.944 (1.472)	14.934 (4.802)	0.9764 (W)	-1.07	Down	15.226 (4.800)	12.593 (4.406)	0.2199	1.21	Up	13.528 (7.248)	15.780 (10.288)	0.5727 (W)	-1.17	Down
2-Oxoglutaric acid	10.299 (7.373)	9.197 (3.881)	0.8823 (W)	1.12	Up	13.336 (9.044)	7.428 (3.712)	0.0622 (W)	1.8	Up	15.567 (6.967)	11.244 (7.556)	0.1229 (W)	1.38	Up
2-Oxoisocaproate	5.979 (3.422)	8.349 (4.568)	0.1963 (W)	-1.4	Down	8.040 (3.492)	5.980 (3.052)	0.0828 (W)	1.34	Up	6.142 (2.508)	9.033 (4.068)	0.1390 (W)	-1.47	Down
3-Hydroxy-3-methylglutaric acid	6.909 (4.338)	9.751 (3.711)	0.1259	-1.41	Down	9.653 (6.380)	7.386 (3.253)	0.435	1.31	Up	11.857 (5.416)	12.522 (6.308)	0.9764 (W)	-1.06	Down
4-Hydroxyphenylacetic acid	7.797 (2.829)	12.036 (9.997)	0.5327 (W)	-1.54	Down	7.968 (5.145)	9.339 (5.077)	0.5683	-1.17	Down	11.873 (12.535)	18.979 (13.147)	0.1756 (W)	-1.6	Down
Acetic acid	165.813 (123.232)	175.555 (173.722)	0.6999 (W)	-1.06	Down	183.630 (203.515)	126.380 (127.162)	0.5327 (W)	1.45	Up	261.225 (261.481)	255.389 (216.192)	0.6140 (W)	1.02	Up

Adipic acid	66.451 (15.532)	87.581 (24.096)	0.0558	-1.32	Down	71.075 (23.153)	69.233 (15.465)	0.8823 (W)	1.03	Up	78.942 (40.203)	96.982 (33.539)	0.2425 (W)	-1.23	Down
Citric acid	68.193 (62.556)	26.363 (24.936)	0.0622 (W)	2.59	Up	67.220 (56.622)	25.087 (19.635)	0.0536 (W)	2.68	Up	50.335 (38.797)	71.807 (83.222)	0.7897 (W)	-1.43	Down
Ethylmalonic acid	20.354 (3.893)	36.103 (25.669)	0.1963 (W)	-1.77	Down	21.239 (5.568)	20.790 (8.421)	0.6565 (W)	1.02	Up	32.907 (17.621)	35.047 (19.209)	0.6999 (W)	-1.07	Down
Formic acid	19.926 (10.166)	16.258 (5.712)	0.2635	1.23	Up	17.218 (6.420)	14.360 (5.011)	0.2612	1.2	Up	13.901 (3.257)	15.382 (7.135)	0.8823 (W)	-1.11	Down
Glyceric acid	34.234 (22.286)	19.182 (9.062)	0.1619	1.78	Up	26.865 (16.858)	15.428 (6.457)	0.16	1.74	Up	21.870 (5.382)	17.479 (12.440)	0.1082 (W)	1.25	Up
Glycolic acid	107.723 (122.265)	146.271 (457.951)	0.3875 (W)	-1.36	Down	26.898 (11.762)	30.571 (17.231)	0.6999 (W)	-1.14	Down	133.673 (55.879)	96.566 (136.661)	0.0536 (W)	1.38	Up
Hippuric acid	137.806 (150.713)	88.799 (47.744)	0.6999 (W)	1.55	Up	66.544 (19.403)	55.034 (18.857)	0.2048	1.21	Up	139.884 (85.142)	107.234 (44.397)	0.4021	1.3	Up
Methylmalonic acid	23.203 (10.512)	21.961 (6.321)	0.7213	1.06	Up	22.241 (6.287)	16.399 (5.510)	0.0233 (W)	1.36	Up	22.101 (8.635)	26.179 (12.136)	0.8358 (W)	-1.18	Down
Succinic acid	101.099 (60.231)	115.391 (43.448)	0.2954 (W)	-1.14	Down	84.647 (24.985)	81.426 (27.992)	0.8027	1.04	Up	137.242 (49.185)	118.860 (62.853)	0.3551 (W)	1.15	Up
trans-Aconitic acid	24.306 (12.585)	18.539 (9.914)	0.2508	1.31	Up	27.093 (19.098)	15.268 (8.239)	0.1941	1.77	Up	19.446 (13.102)	23.955 (21.524)	0.5727 (W)	-1.23	Down
**Alcohols**															
Ethanol	51.493 (23.780)	61.878 (32.309)	0.6140 (W)	-1.2	Down	39.357 (13.041)	42.796 (12.548)	0.2185 (W)	-1.09	Down	40.636 (7.542)	56.190 (22.208)	0.1565 (W)	-1.38	Down
Propylene glycol	33.331 (6.270)	42.388 (22.863)	0.1263	-1.27	Down	34.353 (12.083)	34.344 (14.779)	0.9989	1	Up	31.752 (12.974)	47.599 (29.134)	0.1565 (W)	-1.5	Down
**Misc**															
1,7-Dimethylxanthine	20.044 (35.659)	10.120 (6.809)	0.4942 (W)	1.98	Up	3.296 (1.841)	4.937 (3.452)	0.6999 (W)	-1.5	Down	22.719 (20.191)	14.650 (10.813)	0.6140 (W)	1.55	Up
Creatinine[4]	4039.620 (1860.974)	4723.310 (1621.231)	0.389	-1.17	Down	4060.483 (2160.656)	5024.240 (1519.214)	0.2279	-1.24	Down	7045.717 (3357.020)	4984.760 (2670.551)	0.1963 (W)	1.41	Up
Dimethylamine	43.943 (24.738)	57.231 (70.490)	0.4570 (W)	-1.3	Down	83.897 (111.182)	29.543 (35.629)	0.7445 (W)	2.84	Up	11.240 (6.002)	42.322 (103.940)	0.0536 (W)	-3.77	Down
N-Carbamoyl-beta-alanine	11.668 (4.412)	16.601 (9.103)	0.1565 (W)	-1.42	Down	11.440 (3.626)	12.230 (3.164)	0.6082	-1.07	Down	19.377 (5.612)	26.633 (16.675)	0.6140 (W)	-1.37	Down
Trimethylamine N-oxide	1.966 (1.217)	1.999 (0.888)	0.6565 (W)	-1.02	Down	2.107 (1.012)	1.914 (2.252)	0.1963 (W)	1.1	Up	2.623 (3.072)	1.970 (1.754)	0.7897 (W)	1.33	Up

[1]Cows were diagnosed with ketosis (n=6) ranging from wk +1 to +3.

[2]Only metabolites unique to NMR are shown. More metabolites were measured but because their concentrations were also measured by DI/LC-MS/MS and GC-MS and were not found to be statistically different, these data are not given here.

[3]*p*-value is calculated with t-test as a default, *p*-value with (W) is calculated by the Wilcoxon Mann Whitney test.

[4]Concentration of metabolite (Mean ± SD) is expressed by µM.

Suppl. Table 4. Concentrations of urine metabolites (mean (SD)) in healthy control (CON) and ketotic cows at +4, and +8 wks after parturition as determined by NMR

	4 week after parturition					8 week after parturition				
Metabolite[1], µM/mM creatinine	Ketosis	CON	*P*-value[2]	Fold change	Ketosis / CON	Ketosis	CON	*P*-value	Fold change	Ketosis / CON
Number of cases	6	6	-	-	-	6	6	-	-	-
**Ketones**										
3-Hydroxybutyric acid	458.375 (580.927)	169.260 (325.217)	0.4848 (W)	2.71	Up	307.163 (554.574)	34.096 (22.741)	0.6991 (W)	9.01	Up
Acetoacetic acid	94.240 (100.092)	41.965 (65.668)	0.1320 (W)	2.25	Up	137.534 (213.312)	27.261 (23.897)	0.3095 (W)	5.05	Up
Acetone	327.638 (467.313)	81.026 (174.335)	0.0931 (W)	4.04	Up	250.120 (539.194)	12.392 (6.634)	0.2403 (W)	20.18	Up
**Saccharides**										
1,3-Dihydroxyacetone (DHA)	16.200 (10.741)	6.069 (2.767)	0.0692	2.67	Up	17.963 (9.112)	6.169 (4.140)	0.0162	2.91	Up
Arabinose	38.842 (17.822)	53.044 (26.873)	0.306	-1.37	Down	53.004 (38.465)	32.678 (20.232)	0.2786	1.62	Up
D-Galactose	64.158 (106.494)	37.044 (27.499)	0.6991 (W)	1.73	Up	33.153 (13.122)	246.373 (510.047)	0.3095 (W)	-7.43	Down
D-Glucose	127.762 (148.017)	55.679 (36.293)	0.3095 (W)	2.29	Up	66.666 (32.022)	62.177 (22.973)	0.9372 (W)	1.07	Up
D-Xylose	16.240 (8.192)	14.854 (7.864)	1.0000 (W)	1.09	Up	13.770 (10.470)	12.049 (4.633)	0.5887 (W)	1.14	Up
Lactose	473.724 (1011.324)	30.325 (21.761)	0.2403 (W)	15.62	Up	307.925 (647.753)	107.515 (158.526)	1.0000 (W)	2.86	Up
**Amino acids and derivatives**										
2-Aminobutyric acid	24.534 (11.156)	26.386 (13.520)	0.8011	-1.08	Down	23.357 (18.873)	22.306 (13.585)	0.6991 (W)	1.05	Up
3-Aminoisobutyric acid	49.654 (24.905)	31.239 (16.369)	0.1611	1.59	Up	36.201 (32.624)	31.653 (18.139)	0.6991 (W)	1.14	Up
beta-Alanine	34.495 (21.164)	17.193 (4.290)	0.1026	2.01	Up	19.504 (14.064)	22.150 (22.758)	0.8182 (W)	-1.14	Down
Betaine	140.345 (212.829)	57.317 (63.348)	0.8182 (W)	2.45	Up	60.447 (54.063)	33.263 (28.154)	0.3003	1.82	Up
Creatine	916.915 (574.519)	687.648 (411.988)	0.4454	1.33	Up	806.056 (441.587)	752.553 (353.573)	0.8215	1.07	Up
Guanidoacetic acid	76.454 (25.837)	83.202 (29.908)	0.3939 (W)	-1.09	Down	75.614 (35.921)	76.209 (38.452)	0.9785	-1.01	Down
L-Alloisoleucine	29.857 (18.764)	19.294 (19.544)	0.3095 (W)	1.55	Up	17.382 (10.060)	16.943 (6.302)	0.9296	1.03	Up
L-Cysteine	35.138 (27.772)	20.105 (13.032)	0.3939 (W)	1.75	Up	23.863 (20.497)	23.391 (21.096)	0.6991 (W)	1.02	Up
L-Isoleucine	138.086 (158.591)	61.353 (40.151)	0.1320 (W)	2.25	Up	104.115 (65.755)	57.848 (13.017)	0.0931 (W)	1.8	Up
L-Leucine	258.605 (128.993)	244.645 (122.971)	0.8517	1.06	Up	342.210 (109.620)	294.526 (132.560)	0.5125	1.16	Up
L-Lysine	20.653 (7.755)	11.039 (8.507)	0.068	1.87	Up	13.044 (9.380)	5.792 (3.975)	0.1320 (W)	2.25	Up
L-Methionine	32.400 (24.035)	23.740 (27.313)	0.5728	1.36	Up	39.564 (53.393)	24.299 (30.510)	0.4848 (W)	1.63	Up
L-Phenylalanine	23.680 (12.933)	16.650 (10.146)	0.1797 (W)	1.42	Up	17.653 (5.000)	12.330 (8.927)	0.1320 (W)	1.43	Up
L-Tyrosine	28.370 (21.923)	36.057 (28.075)	0.6991 (W)	-1.27	Down	28.033 (11.419)	32.253 (13.445)	0.5708	-1.15	Down
L-Valine	2318.793 (949.790)	1260.075 (805.155)	0.0639	1.84	Up	2630.603 (1580.904)	1473.110 (1077.430)	0.0931 (W)	1.79	Up
N,N-Dimethylglycine	23.612 (7.598)	26.213 (13.616)	0.6914	-1.11	Down	24.660 (13.375)	21.196 (7.577)	0.5932	1.16	Up

N-Acetylaspartic acid	13.630 (25.738)	6.529 (7.572)	0.3939 (W)	2.09	Up	5.152 (6.583)	9.800 (9.043)	0.5887 (W)	-1.9	Down
N-Acetylglutamic acid	31.575 (19.729)	11.275 (3.833)	0.0022 (W)	2.8	Up	15.179 (2.822)	17.305 (9.756)	0.6269	-1.14	Down
N-Acetylglutamine	56.817 (18.253)	43.822 (27.950)	0.3628	1.3	Up	38.877 (21.317)	26.176 (11.702)	0.2296	1.49	Up
N-Phenylacetylglycine	66.421 (31.827)	74.650 (35.840)	0.683	-1.12	Down	56.665 (9.821)	49.978 (17.265)	0.4288	1.13	Up
Pantothenic acid	4.420 (4.109)	2.488 (1.307)	0.3144	1.78	Up	3.662 (2.422)	3.942 (3.671)	0.879	-1.08	Down
Sarcosine	52.575 (37.514)	25.714 (14.516)	0.1329	2.04	Up	42.940 (32.915)	36.055 (35.106)	0.9372 (W)	1.19	Up
Tiglylglycine	18.367 (9.831)	10.873 (6.744)	0.1547	1.69	Up	13.714 (7.195)	12.457 (12.827)	0.3095 (W)	1.1	Up
1-Methylhistidine	17.345 (18.874)	11.297 (8.905)	0.4848 (W)	1.54	Up	10.697 (3.862)	6.655 (4.902)	0.1437	1.61	Up
3-Methylhistidine	34.672 (25.549)	24.467 (9.084)	0.3908	1.42	Up	34.131 (20.375)	24.419 (18.383)	0.3095 (W)	1.4	Up
**Organic acids**										
1,3-Dimethyluric acid	13.885 (5.446)	14.066 (5.616)	0.956	-1.01	Down	13.092 (8.036)	10.157 (8.345)	0.2403 (W)	1.29	Up
2-Hydroxy-3-methylpentanoic acid	146.632 (30.268)	129.480 (68.289)	0.5862	1.13	Up	122.132 (57.797)	114.619 (54.392)	0.8213	1.07	Up
2-Hydroxybutyric acid	85.432 (59.207)	67.727 (33.528)	0.4848 (W)	1.26	Up	70.360 (71.978)	53.367 (22.752)	0.6991 (W)	1.32	Up
2-Hydroxyisobutyric acid	9.887 (4.070)	9.255 (4.176)	0.7959	1.07	Up	7.432 (3.823)	10.290 (8.970)	0.5887 (W)	-1.38	Down
2-Hydroxyvaleric acid	140.021 (73.492)	97.868 (41.720)	0.2498	1.43	Up	118.579 (68.679)	90.468 (34.577)	0.3916	1.31	Up
2-Methylglutaric acid	54.192 (44.309)	38.498 (35.735)	0.3939 (W)	1.41	Up	45.759 (38.856)	34.453 (38.741)	0.4848 (W)	1.33	Up
2-Oxobutyric acid	31.987 (26.416)	22.689 (10.312)	1.0000 (W)	1.41	Up	28.204 (29.676)	17.707 (9.350)	0.9372 (W)	1.59	Up
2-Oxoglutaric acid	29.671 (29.784)	23.626 (12.049)	0.8182 (W)	1.26	Up	15.904 (11.569)	18.175 (12.432)	0.4848 (W)	-1.14	Down
2-Oxoisocaproate	12.674 (4.802)	9.475 (6.682)	0.3634	1.34	Up	8.936 (2.909)	8.804 (6.111)	0.3939 (W)	1.02	Up
3-Hydroxy-3-methylglutaric acid	19.834 (11.183)	15.980 (8.255)	0.5125	1.24	Up	10.358 (4.161)	10.073 (4.879)	0.9154	1.03	Up
4-Hydroxyphenylacetic acid	49.293 (36.078)	28.858 (51.413)	0.0931 (W)	1.71	Up	34.317 (33.565)	11.142 (18.002)	0.0931 (W)	3.08	Up
Acetic acid	1411.596 (1649.546)	772.016 (1283.069)	0.3939 (W)	1.83	Up	713.494 (593.061)	136.268 (97.716)	0.0627	5.24	Up
Adipic acid	120.652 (37.307)	111.790 (38.864)	0.6955	1.08	Up	102.706 (45.099)	95.081 (16.690)	0.8182 (W)	1.08	Up
Ascorbic acid	20.527 (15.308)	18.237 (8.166)	0.8182 (W)	1.13	Up	13.545 (3.598)	19.285 (9.603)	0.0931 (W)	-1.42	Down
Citric acid	163.426 (236.465)	157.503 (141.950)	0.9372 (W)	1.04	Up	38.976 (35.855)	39.861 (22.074)	0.6991 (W)	-1.02	Down
Ethylmalonic acid	47.775 (20.340)	43.790 (25.927)	0.7731	1.09	Up	37.771 (29.530)	92.704 (154.726)	0.3939 (W)	-2.45	Down
Formic acid	26.622 (9.592)	22.530 (8.359)	0.4491	1.18	Up	24.820 (9.690)	24.472 (15.950)	0.8182 (W)	1.01	Up
Gluconic acid	14.882 (11.856)	17.467 (12.953)	0.7258	-1.17	Down	18.921 (9.173)	10.635 (9.230)	0.1499	1.78	Up
Glyceric acid	52.085 (44.930)	25.973 (11.272)	0.1797 (W)	2.01	Up	36.208 (13.792)	23.340 (12.075)	0.1163	1.55	Up
Glycolic acid	1572.371 (3430.254)	62.492 (25.274)	0.3095 (W)	25.16	Up	561.988 (809.047)	77.907 (29.028)	0.0411 (W)	7.21	Up
Hippuric acid	101.948 (20.830)	99.843 (24.451)	0.6991 (W)	1.02	Up	99.687 (36.872)	93.433 (32.135)	0.7606	1.07	Up
Isocitric acid	44.605 (41.802)	30.728 (5.871)	0.9372 (W)	1.45	Up	26.702 (8.706)	27.659 (9.939)	0.8627	-1.04	Down

L-Lactic acid	21.743 (17.645)	14.643 (11.379)	0.4268	1.48	Up	13.674 (7.863)	7.813 (4.528)	0.1447	1.75	Up
Methylmalonic acid	20.526 (6.506)	31.021 (8.295)	0.0349	-1.51	Down	26.946 (11.666)	28.593 (13.972)	0.8291	-1.06	Down
Succinic acid	133.553 (59.044)	117.216 (58.755)	0.6413	1.14	Up	130.391 (66.373)	149.608 (22.961)	0.0649 (W)	-1.15	Down
trans-Aconitic acid	46.754 (52.995)	26.538 (15.544)	0.5887 (W)	1.76	Up	24.384 (12.492)	24.387 (10.671)	0.9997	-1	Down
**Alcohols**										
Ethanol	138.203 (196.290)	86.663 (80.363)	0.4848 (W)	1.59	Up	100.213 (54.155)	74.614 (30.616)	0.3372	1.34	Up
Methanol	49.606 (28.921)	24.941 (15.264)	0.0944	1.99	Up	38.791 (25.085)	28.947 (16.013)	0.3095 (W)	1.34	Up
Propylene glycol	69.048 (60.574)	43.005 (30.200)	0.3682	1.61	Up	61.499 (61.995)	32.298 (22.223)	0.3095 (W)	1.9	Up
**Misc**										
1,7-Dimethylxanthine	10.119 (5.534)	16.429 (7.387)	0.125	-1.62	Down	7.765 (6.763)	12.470 (6.280)	0.2402	-1.61	Down
3-Indoxyl sulfate	147.618 (87.275)	100.128 (71.689)	0.3273	1.47	Up	109.685 (88.476)	79.611 (55.363)	0.9372 (W)	1.38	Up
Creatinine[3]	3440.867 (1251.254)	3914.033 (1707.166)	0.596	-1.14	Down	3560.017 (1146.611)	4645.567 (1458.799)	0.1824	-1.3	Down
Dimethyl sulfone	112.708 (65.829)	97.621 (42.540)	0.8182 (W)	1.15	Up	99.685 (31.659)	76.055 (24.768)	0.1804	1.31	Up
Dimethylamine	29.117 (16.432)	29.795 (27.579)	0.6991 (W)	-1.02	Down	29.861 (22.231)	23.005 (13.868)	0.536	1.3	Up
Hypoxanthine	53.963 (35.430)	39.390 (20.109)	0.6991 (W)	1.37	Up	49.565 (53.054)	32.644 (22.343)	0.8182 (W)	1.52	Up
Imidazole	5.324 (4.902)	3.119 (1.232)	0.5887 (W)	1.71	Up	3.875 (1.365)	5.024 (3.633)	0.4849	-1.3	Down
myo-Inositol	48.302 (37.558)	26.803 (15.344)	0.1320 (W)	1.8	Up	33.364 (16.568)	29.693 (17.934)	0.7204	1.12	Up
N-Carbamoyl-beta-alanine	23.298 (7.163)	19.985 (6.976)	0.4359	1.17	Up	19.794 (5.525)	16.087 (4.201)	0.22	1.23	Up
O-Phosphocholine	159.304 (67.607)	128.349 (92.295)	0.3095 (W)	1.24	Up	133.480 (87.832)	101.820 (39.175)	0.4388	1.31	Up
Trimethylamine	66.894 (17.338)	69.675 (72.077)	0.3095 (W)	-1.04	Down	49.085 (15.591)	45.954 (27.989)	0.8156	1.07	Up
Trimethylamine N-oxide	77.585 (185.058)	1.578 (0.745)	0.2403 (W)	49.16	Up	2.197 (1.528)	6.823 (13.192)	1.0000 (W)	-3.11	Down
Uracil	55.867 (64.521)	26.955 (13.021)	0.3939 (W)	2.07	Up	39.823 (28.331)	21.478 (12.106)	0.1320 (W)	1.85	Up
Urea	62.431 (51.310)	39.379 (18.851)	0.3395	1.59	Up	61.897 (31.577)	51.388 (28.931)	0.8182 (W)	1.2	Up

[1]Only metabolites unique to NMR are shown. More metabolites were measured but because their concentrations were also measured by DI/LC-MS/MS and GC-MS and were not found to be statistically different, these data are not given here.

[2]*p*-value is calculated with t-test as a default, *p*-value with (W) is calculated by the Wilcoxon Mann Whitney test.

[3]Concentration of metabolite (Mean ± SD) is expressed by μM.

Suppl. Table 5. Concentrations of non-siginficant urine metabolites (mean (SD)) in healthy control (CON) and ketotic cows at 3 time points (-8 wk, -4 wk, and the wk of diagnosis of disease) as determined by GC-MS

	8 week before parturition					4 week before parturition					Ketosis diagnosis week[1]				
Metabolite[2], μM/mM creatinine	Ketosis	CON	*P*-value[3]	Fold change	Ketosis / CON	Ketosis	CON	*P*-value	Fold change	Ketosis / CON	Ketosis	CON	*P*-value	Fold change	Ketosis / CON
Number of cases	6	20	-	-	-	6	20	-	-	-	6	20	-	-	
Pyruvic acid	0.062 (0.029)	0.050 (0.028)	0.1130 (W)	1.24	Up						0.046 (0.000)	0.046 (0.022)	0.1868 (W)	1	Up
Malonic acid	0.107 (0.043)	0.147 (0.069)	0.0737 (W)	-1.38	Down						0.135 (0.081)	0.202 (0.156)	0.6308 (W)	-1.49	Down
3-Hydroxyisovaleric acid	0.272 (0.169)	0.472 (0.544)	0.8049 (W)	-1.73	Down						0.917 (0.452)	1.224 (1.116)	0.7325 (W)	-1.34	Down
Adipic acid	0.007 (0.001)	0.007 (0.008)	0.5078 (W)	-1	Down	0.003 (0.002)	0.003 (0.004)	0.5227 (W)	-1	Down	0.008 (0.005)	0.010 (0.006)	0.7780 (W)	-1.22	Down
Pimelic acid	0.021 (0.013)	0.026 (0.020)	0.6194 (W)	-1.21	Down	0.010 (0.009)	0.013 (0.015)	0.8791 (W)	-1.29	Down	0.013 (0.005)	0.012 (0.010)	0.6411 (W)	1.02	Up
m-Hydroxyphenylacetic acid	0.009 (0.004)	0.006 (0.004)	0.0713 (W)	1.47	Up	0.005 (0.001)	0.005 (0.003)	0.9500 (W)	-1.1	Down	0.007 (0.006)	0.014 (0.017)	0.2005 (W)	-2.17	Down
p-Hydroxyphenylacetic acid	0.050 (0.022)	0.040 (0.030)	0.4162 (W)	1.24	Up	0.018 (0.018)	0.032 (0.032)	0.4112 (W)	-1.8	Down	0.051 (0.002)	0.052 (0.031)	1.0000 (W)	-1.02	Down
Suberic acid	0.023 (0.016)	0.015 (0.015)	0.0709 (W)	1.55	Up	0.010 (0.006)	0.017 (0.017)	0.4587 (W)	-1.69	Down	0.011 (0.005)	0.019 (0.022)	0.7609 (W)	-1.74	Down
Sebacic acid	0.034 (0.038)	0.020 (0.009)	0.3473 (W)	1.71	Up	0.007 (0.004)	0.026 (0.033)	0.0726 (W)	-3.95	Down	0.026 (0.011)	0.037 (0.030)	0.3308 (W)	-1.43	Down
Phenaceturic acid	0.138 (0.072)	0.197 (0.085)	0.0957 (W)	-1.43	Down	0.120 (0.179)	0.167 (0.328)	0.9273 (W)	-1.39	Down	0.343 (0.104)	0.398 (0.213)	0.5642 (W)	-1.16	Down
Hydroxyphenyllactic acid	0.004 (0.003)	0.008 (0.008)	0.1743 (W)	-1.85	Down	0.005 (0.003)	0.004 (0.005)	0.2868 (W)	1.1	Up	0.011 (0.006)	0.015 (0.013)	0.5106 (W)	-1.44	Down
m-Hydroxyhippuric acid	0.016 (0.004)	0.016 (0.006)	0.9741 (W)	1.05	Up	0.009 (0.007)	0.017 (0.031)	0.9273 (W)	-1.84	Down	0.012 (0.003)	0.014 (0.009)	0.9755 (W)	-1.18	Down
Stearic acid	0.027 (0.020)	0.030 (0.042)	0.4570 (W)	-1.12	Down	0.020 (0.013)	0.021 (0.016)	0.9515 (W)	-1.01	Down	0.036 (0.039)	0.029 (0.029)	0.9764 (W)	1.24	Up

[1]Cows were diagnosed with ketosis (n=6) ranging from wk +1 to +3.

[2]Only metabolites unique to GC-MS are shown. More metabolites were measured but because their concentrations were also measured by NMR and DI/LC-MS/MS and were not found to be statistically different, these data are not given here.

[3]*p*-value is calculated with t-test as a default, *p*-value with (W) is calculated by the Wilcoxon Mann Whitney test.

Suppl. Table 6. Concentrations of urine metabolites (mean (SD)) in healthy control (CON) and ketotic cows at +4, and +8 wks after parturition as determined by GC-MS

	4 week after parturition					8 week after parturition				
Metabolite[1], μM/mM creatinine	Ketosis	CON	*P*-value[2]	Fold change	Ketosis / CON	Ketosis	CON	*P*-value	Fold change	Ketosis / CON
Number of cases	6	6	-	-	-	6	6	-	-	-
Pyruvic acid										
Malonic acid						0.074 (0.030)	0.099 (0.070)	1.0000 (W)	-1.33	Down
3-Hydroxyisovaleric acid										
Adipic acid	0.036 (0.024)	0.066 (0.077)	0.6553 (W)	-1.84	Down	0.010 (0.006)	0.017 (0.013)	0.3700 (W)	-1.77	Down
Pimelic acid	0.049 (0.029)	0.087 (0.086)	0.6553 (W)	-1.76	Down	0.022 (0.013)	0.038 (0.035)	0.8068 (W)	-1.75	Down
m-Hydroxyphenylacetic acid	0.041 (0.031)	0.098 (0.118)	0.3611 (W)	-2.36	Down	0.020 (0.012)	0.040 (0.045)	0.3261	-2.01	Down
p-Hydroxyphenylacetic acid	0.159 (0.029)	0.175 (0.044)	0.9241 (W)	-1.11	Down	0.078 (0.046)	0.066 (0.032)	0.615	1.18	Up
Suberic acid	0.015 (0.014)	0.055 (0.070)	0.1255 (W)	-3.75	Down	0.006 (0.007)	0.033 (0.024)	0.0087 (W)	-5.54	Down
Azelaic acid	0.024 (0.028)	0.079 (0.072)	0.1139	-3.27	Down	0.023 (0.027)	0.120 (0.093)	0.0508	-5.21	Down
Sebacic acid	0.033 (0.001)	0.034 (0.018)	0.7526 (W)	-1.03	Down					
Phenaceturic acid	0.142 (0.019)	0.126 (0.019)	0.2184 (W)	1.12	Up					
Hydroxyphenyllactic acid	0.018 (0.005)	0.023 (0.025)	0.8489 (W)	-1.24	Down					
m-Hydroxyhippuric acid	0.010 (0.002)	0.009 (0.002)	0.2184 (W)	1.18	Up					
Stearic acid	0.024 (0.013)	0.025 (0.015)	0.9264	-1.03	Down	0.020 (0.009)	0.068 (0.091)	0.1320 (W)	-3.3	Down

[1]Only metabolites unique to ICP-MS and GC-MS are shown. More metabolites were measured but because their concentrations were also measured by NMR and DI/LC-MS/MS and were not found to be statistically different, these data are not given here.

[2]*p*-value is calculated with t-test as a default, *p*-value with (W) is calculated by the Wilcoxon Mann Whitney test.
